# Cross-Tissue Regulatory Network Analyses Reveal Novel Susceptibility Genes and Potential Mechanisms for Endometriosis

**DOI:** 10.3390/biology13110871

**Published:** 2024-10-26

**Authors:** Mingrui Zou, Mingmei Lin, Kai-Lun Hu, Rong Li

**Affiliations:** 1 Center for Reproductive Medicine, Department of Obstetrics and Gynecology, Peking University Third Hospital, Beijing 100191, China; 2110301145@stu.pku.edu.cn (M.Z.); linmingmei2023@163.com (M.L.); 2 National Clinical Research Center for Obstetrics and Gynecology, Peking University Third Hospital, Beijing 100191, China; 3 Key Laboratory of Assisted Reproduction, Peking University, Ministry of Education, Beijing 100191, China; 4 Beijing Key Laboratory of Reproductive Endocrinology and Assisted Reproductive Technology, Beijing 100191, China; 5 Peking University First School of Clinical Medicine, Peking University First Hospital, Beijing 100034, China

**Keywords:** colocalization, endometriosis, GWAS, Mendelian randomization, TWAS

## Abstract

Endometriosis (EMT) is a common gynecological disease with a strong genetic component, while its precise etiology remains elusive. This study aims to integrate transcriptome-wide association study (TWAS), Mendelian randomization (MR), and bioinformatics analyses to reveal novel putatively causal genes and potential mechanisms. We obtained summary-level data of the Genotype-Tissue Expression Project (GTEx), v8 expression quantitative loci (eQTL) data, and the genome-wide association study (GWAS) data of EMT and its subtypes from the R11 release results of the FinnGen consortium for analysis. GWAS data of modifiable risk factors were collected from IEU Open GWAS. Cross-tissue TWAS analyses were performed using the unified test for molecular signature (UTMOST), while functional summary-based imputation (FUSION) was employed for single-tissue TWAS analyses. Furthermore, we also conducted multi-marker analysis of genomic annotation (MAGMA) analyses to validate the significant associations. Subsequent Mendelian randomization (MR) and colocalization analysis elucidated the causal associations between the identified genes across various tissues and EMT. To further delve into mechanisms, two-sample network MR analyses were conducted. At last, bioinformatics analyses were employed to enhance our understanding of the functional implications and expression patterns of these identified genes. For EMT, 22 significant gene signals were identified by UTMOST, 615 by FUSION, and 354 by MAGMA. Ultimately, six genes, including CISD2, EFRB, GREB1, IMMT, SULT1E1, and UBE2D3, were identified as candidate susceptibility genes for EMT. Through similar procedures, we identified GREB1, IL1A, and SULT1E1 for EMT of the ovary, and we identified GREB1 for EMT of the pelvic peritoneum, EMT of rectovaginal septum and vagina, and deep EMT. In MR analyses, the expression of IMMT in 21 tissues, EFR3B in the adrenal gland, CISD2 in 17 tissues, and UBE2D3 in 7 tissues demonstrated causal relationships with EMT risk. In addition, CISD2, IMMT, and UBE2D3, across different tissues, exhibited strong colocalization with EMT (PPH4 > 0.7). Two-sample network MR analyses revealed that CISD2, EFR3B, and UBE2D3 could potentially regulate the levels of blood lipids and hip circumference so as to influence the risk of EMT. Furthermore, bioinformatics analyses confirmed our findings and delved into the biological functions of the identified genes. Our study unveiled seven novel candidate genes whose predicted expression was associated with the risk of EMT, providing new insights into the underlying genetic framework of EMT. These findings will facilitate a deeper comprehension of the tissue-specific transcriptional regulatory mechanisms associated with EMT, paving the way for optimizing the management and treatment of EMT.

## Simple Summary

Endometriosis (EMT) is a chronic gynecological disease affecting millions of women. Nevertheless, the precise mechanisms underlying this disorder remain largely unexplored. In this study, we conducted transcriptome-wide association study (TWAS) analyses to uncover novel susceptibility genes linked to EMT. In addition, we employed Mendelian randomization (MR) and colocalization analyses to delve into the causal associations between candidate genes across different tissues and EMT. Moreover, to elucidate the mechanisms by which these genes influence the risk of EMT, we conducted two-sample network MR analyses to assess the mediating roles of modifiable risk factors in the causal pathways connecting identified genes across tissues and EMT. Our findings revealed that expression levels of several genes, including CISD2, EFRB, GREB1, IMMT, SULT1E1, and UBE2D3, across various tissues influenced the risk of EMT, with blood lipids levels and hip circumference serving as mediators in these associations. These findings contribute to a deeper understanding of the tissue-specific transcriptional regulatory mechanisms associated with EMT, offering insights that may enhance the management and treatment strategies for EMT.

## 1. Introduction

Endometriosis (EMT) is a chronic gynecological condition characterized by the presence of endometrial-like tissue outside the uterine cavity, leading to a spectrum of clinical manifestations, including pelvic pain, dysmenorrhea, and infertility [1]. Statistics from Katholieke University indicate that EMT affects 5–10% of women of reproductive age (approximately 176 million women worldwide), with an economic burden surpassing USD 22 billion in the USA alone [2]. Additionally, there is a notable deficiency in the recognition regarding EMT, as the diagnosis times vary from 4 to 11 years, with nearly 65% of women facing initial misdiagnosis [3,4]. At present, the etiology of EMT is not yet fully elucidated, and conventional pharmaceutical interventions often provide merely symptomatic relief without addressing the fundamental pathophysiological mechanisms of the condition [5]. Although surgical interventions may yield positive outcomes, the risk of recurrence remains significantly high [6]. Therefore, acquiring a deep understanding of susceptibility genes and the underlying biological mechanisms of EMT will contribute to the development of effective therapeutic targets.

According to previous studies, EMT has a substantial genetic component, with heritability estimated at around 50% [7]. Thus, uncovering novel genetic variants strongly associated with EMT risk could provide valuable insights into its pathogenesis. In recent years, numerous genome-wide association studies (GWAS) have been conducted to identify genetic risk loci for EMT. For instance, a large GWAS meta-analysis involving 17,045 EMT patients and 191,596 controls confirmed 14 genetic loci robustly linked to EMT. However, these loci only explained 1.75% of total EMT risk variance [8]. Furthermore, limited statistical power, complex linkage disequilibrium (LD), and tissue-specific regulatory mechanisms make it difficult for GWAS to identify risk loci and candidate genes accurately, and biological interpretation and functional comprehension also remain elusive [9,10]. Consequently, it is imperative to introduce novel analytical strategies to enhance the identification of risk loci and susceptibility genes associated with EMT.

The transcriptome-wide association study (TWAS) is a novel genetic association analysis strategy which integrates expression quantitative trait loci (eQTL) and GWAS data to identify novel susceptibility genes. This approach employs an externally trained inference model that utilizes genotypes to infer gene expression levels and investigate their association with particular traits of interest [11]. Furthermore, considering the tissue-dependent nature of transcriptional regulation and their resemblance in transcriptional regulation across tissues, a cross-tissue TWAS strategy known as the unified test for molecular signature (UTMOST) emerges [12]. Compared to single-tissue TWAS, this approach enhances the precision and efficacy of the imputation models by applying a group lasso penalty, facilitating the identification of shared cross-tissue eQTL effects alongside the preservation of robust tissue-specific eQTL effects [13]. As is well known, in real life, due to ethical constraints, we are often unable to study the associations between gene expression levels in various organs of the body and a certain disease. However, with the rise of cross-tissue TWAS analysis, we can attempt to explore the transcriptional regulation patterns of diseases between different organs. In recent years, cross-tissue TWAS analysis has been widely used to discover novel susceptibility genes for diseases such as rheumatoid arthritis [13], migraine [14], and lung cancer [15], whose biologically relevant tissues remain somewhat elusive. Nevertheless, the transcriptional regulatory patterns across tissues of EMT are still unclear.

**Figure 1:**
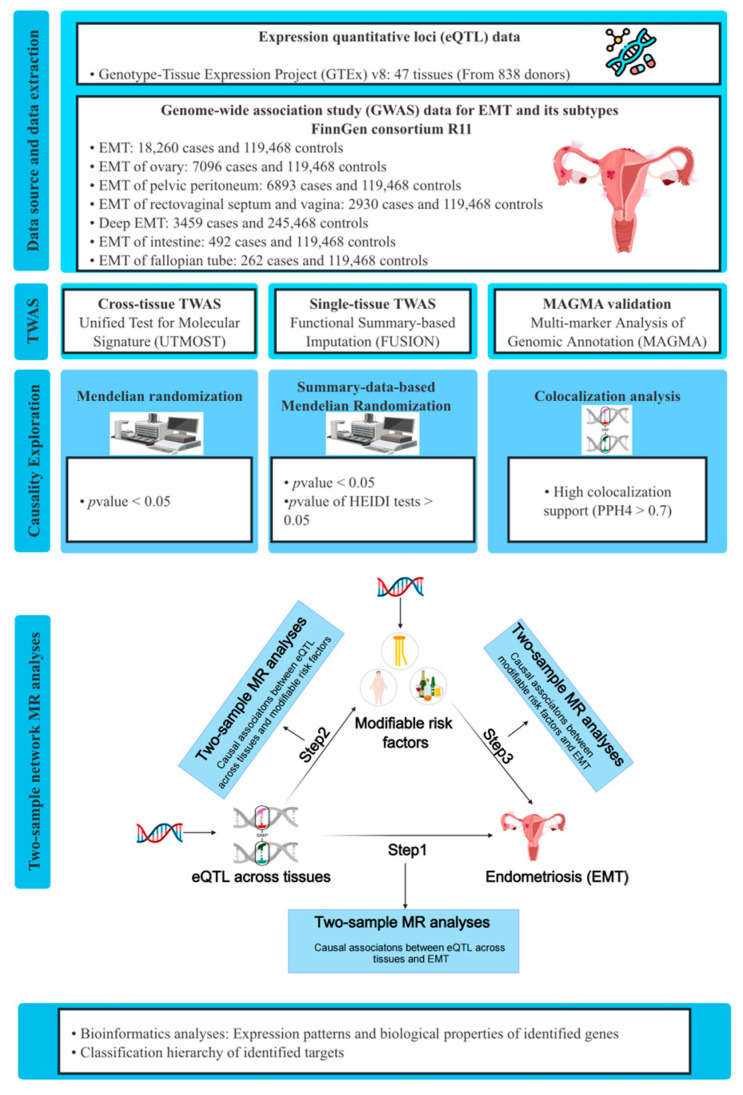
The study design of this investigation. GWAS, genome-wide association studies; GTEx, Genotype-Tissues Expression Project; TWAS, transcriptome-wide association studies; UTMOST, unified test for molecular signatures; FUSION, functional summary-based imputation; MAGMA, multi-marker analysis of GenoMic annotation; eQTL, expression quantitative trait loci; EMT, endometriosis; HEIDI, heterogeneity in dependent instruments; PPH4, posterior probability of hypothesis 4.

In this study, we carried out TWAS analyses by integrating EMT GWAS data from FinnGen R11 with eQTL data from the Genotype-Tissue Expression Project (GTEx) v8. UTMOST was utilized for cross-tissue TWAS analyses, while functional summary-based imputation (FUSION) was employed for single-tissue TWAS analyses [11,12]. Additionally, multi-marker analysis of genomic annotation (MAGMA) analyses were conducted for validation [16]. Subsequently, Mendelian randomization (MR), summary-data-based Mendelian randomization (SMR), and colocalization analyses were performed to delve into the causal associations between candidate genes across different tissues and EMT. To explain the underlying mechanisms how identified genes across various tissues influence the risk of EMT, we conducted two-sample network MR analyses to assess the mediating roles of modifiable risk factors in the associations between identified genes across tissues and EMT. Further bioinformatics analyses revealed the expression patterns and biological properties of the identified genes. The detailed study design is illustrated in Figure 1.

## 2. Materials and Methods

### 2.1. Data Source

GWAS data of EMT were obtained from the FinnGen study R11, which included EMT (18,260 cases and 119,468 controls), EMT of ovary (7096 cases and 119,468 controls), EMT of pelvic peritoneum (6893 cases and 119,468 controls), EMT of rectovaginal septum and vagina (2930 cases and 119,468 controls), deep EMT (3459 cases and 245,468 controls), EMT of intestine (492 cases and 119,468 controls), and EMT of fallopian tube (262 cases and 119,468 controls). EMT and its subtypes from FinnGen consortium were defined by the code N80, N801, N802, N803, N804, N805, and N808 in the International Classification of Diseases, 10th version [17].

The eQTL data from the GTEx v8 dataset, which encompasses a wealth of gene expression data across 49 different tissues, were obtained for analysis [18]. The sample sizes of these data across different tissues range from 73 samples in the kidney cortex to 706 samples in the skeletal muscle. Given that EMT is a gynecological disease, tissues specific to males such as prostate and testis were omitted, and the remaining 47 tissues were included in the analysis.

The GWAS data of modifiable risk factors (anthropometric measurements, common blood indicators, and lifestyle factors) all came from IEU Open GWAS (https://gwas.mrcieu.ac.uk, accessed on 1 August 2024). Anthropometric measurements included body mass index (BMI), waist-hip ratio, waist circumference (WC), hip circumference (HC), height, and weight. Blood indicators included glucose levels, neutrophil count, red blood cell (RBC) count, white blood cell (WBC) count, triglycerides (TG) levels, total cholesterol (TC) levels, very-low-density lipoprotein cholesterol (VLDL-C) levels, low-density lipoprotein cholesterol (LDL-C) levels, high-density lipoprotein cholesterol (HDL-C) levels, apolipoprotein A levels and apolipoprotein B levels. Lifestyle factors included insomnia, alcohol intake frequency, coffee intake, and smoking initiation. The detailed information of these GWAS datasets was displayed in Supplementary Table S1.

### 2.2. Cross-Tissue TWAS Analyses

Firstly, cross-tissue TWAS analyses were performed using the UTMOST method [12]. We integrated the GWAS data of EMT along with the eQTL data of 47 tissues and developed a cross-tissue expression inference model to estimate the gene expression levels within each tissue. Subsequently, a generalized Berk–Jones (GBJ) test was applied to integrate gene–trait associations in 47 tissues based on the covariance from single-tissue statistics [12,19]. We employed the false discovery rate (FDR) method for multiple testing correction, and FDR < 0.05 was considered statistically significant [20].

### 2.3. Single-Tissue TWAS Analyses

Single-tissue TWAS analyses were also conducted using the FUSION method to integrate EMT GWAS data and eQTL data from 47 tissues. FUSION facilitates the development of predictive models of the genetic components of a functional or molecular phenotype. Additionally, it employs GWAS to summarize statistical predictions and evaluate the association between genetic components and disease [11]. For analysis, FUSION constructs multiple predictive models, including BLUP, BSLMM, Elastic Net, GBLUP, and LASSO, to assess the overall effect of single-nucleotide polymorphisms (SNPs) on gene expression weights (the model exhibiting the greatest predictive performance was employed to determine the gene weights) [21]. Subsequently, the genetic effect of EMT and gene weights were combined to perform TWAS analyses. Similar to UTMOST, FDR correction was conducted [20].

### 2.4. Gene-Based Analyses for Validation

We further performed gene-based analyses to validate our findings using the MAGMA software (V.1.10). MAGMA analysis is widely applied in the association analysis of genes and gene sets, which serves to pinpoint functional genes and regulatory pathways associated with target traits, thus standing as a crucial complement to GWAS [16]. Likewise, FDR correction was carried out in MAGMA analysis. The candidate genes included in subsequent MR analysis should meet the following criteria: (1) FDR < 0.05 in cross-tissue TWAS analysis; (2) FDR < 0.05 in at least one tissue in single-tissue TWAS analysis; (3) FDR < 0.05 in MAGMA analysis.

### 2.5. Conditional and Joint Analyses

Conditional and joint (COJO) analyses were performed to identify independent genetic signals (significant results in FUSION analyses), which help us distinguish whether the effect comes from a single SNP or from multiple SNPs working together. Following this analysis, genes that represent independent associations were referred to as jointly significant, while those that no longer showed significance were considered marginally significant. The detailed methods of COJO analyses have been mentioned in previous studies [11,22].

### 2.6. Mendelian Randomization and Colocalization Analyses Between Identified Genes and EMT

To further investigate the causal associations between the identified genes and EMT risk, we conducted Mendelian randomization (MR) and summary-data-based Mendelian randomization (SMR) analyses. The MR analysis is based on 3 fundamental hypotheses: (1) the genetic instrumental variables (IVs) must be robustly associated with the exposure; (2) IVs should be unaffected by confounding factors related to either the exposure or the outcome; (3) IVs are only allowed to influence the outcome through the exposure [23]. For MR analysis, the cis-eQTL of identified genes across different tissues from GTEx v8 were selected as IVs, and the GWAS data of EMT served as outcomes. Only significant (*p* < 5 × 10^−8^), independent (LD clumping r^2^ < 0.001), and strong (F-statistics >10) SNPs were selected as IVs. Palindromic SNPs, as well as those containing missing data, were eliminated. As only one IV was available in each MR analysis, the Wald ratio method was utilized to evaluate the causal effect [24]. MR results are demonstrated in the form of odds ratios (OR) accompanied by 95% confidence intervals (95% CI), with a significant threshold of *p*-value < 0.05. Furthermore, we also performed Steiger filtering analysis to detect reverse causality. If the direction is “TRUE” and the *p*-value < 0.05, reverse causality is considered absent [25]. MR and Steiger filtering analyses were performed using “TwoSampleMR” R package (V.0.5.8) in software R (V.4.3.1).

SMR is a novel form of MR analysis, which is based on the top associated cis-QTL and could reach a higher statistical power than traditional MR analysis [26]. To further augment our findings, SMR analyses were performed. We selected common (minor allele frequency (MAF) > 0.01) cis-eQTLs, which were significantly (*p* < 5 × 10^−8^) associated the expression of identified genes across tissues as IVs. SMR analyses were performed using SMR software (V.1.3.1) [26], and *p*-value < 0.05 was considered statistically significant. In addition, we conducted a heterogeneity in dependent instruments (HEIDI) test to evaluate whether the observed causal association was influenced by the linkage scenario. A *p*-value of the HEIDI test < 0.05 suggested that the association was presumably due to LD [26].

Bayesian colocalization analyses were also applied to enhance the robustness of our findings, which could determine whether the causal effects were driven by LD or a shared genetic variant [24]. The “coloc” R package (V.5.2.3) was used with the following parameters: (1) P_1_ = 1 × 10^−4^: the prior probability of the SNP being associated sorely with trait 1; (2) P_2_ = 1 × 10^−4^: the prior probability of the SNP being associated sorely with trait 2 and (3) P_12_ = 1 × 10^−5^: the prior probability of the SNP being associated with both traits. The Bayesian colocalization analysis assesses the support for five hypotheses: (1) H0: no association with either trait; (2) H1: association only with trait 1; (3) H2: association only with trait 2; (4) H3: association with both traits, but with different causal variants; and (5) H4: association with both traits, sharing a common causal variant. Each hypothesis has a posterior probability (PP). Posterior probability for hypothesis 4 (PPH4) ≥ 0.7 indicates strong evidence of colocalization [27].

### 2.7. Two-Step Network MR Analyses

To further delve into the mechanisms of the associations between the identified genes across tissues and EMT, we conducted two-sample MR analyses to evaluate the causal relationship between the identified genes across tissues and modifiable risk factors, as well as modifiable risk factors and EMT. The strategies used to select IVs for modifiable risk factors were the same as those mentioned above. Finally, 4378 IVs were selected (Supplementary Table S2). The Wald ratio and inverse-variance-weighted (IVW) method served as the primary statistical model [24,28], and MR-Egger regression, weighted median, and weighted mode method were also employed as additional methods [29,30,31]. Moreover, FDR correction was also performed, and strategies including Cochran’s Q test and the MR Egger intercept test were employed to assess potential heterogeneity and horizontal pleiotropy [20,29,32].

Subsequently, two-sample network MR analyses were carried out to assess the mediating roles of modifiable risk factors in the associations between identified genes across tissues and EMT. When there was evidence suggesting that the identified genes across tissues impacted modifiable risk factors, which subsequently influenced the risk of EMT, we would compute the indirect effect (mediating effect) by multiplying the estimated effect of exposure on the mediator with the estimated effect of the mediator on outcome [33]. The standard errors for the indirect effects were derived using the delta method [34]. Furthermore, the proportion mediated by modifiable risk factors was further calculated by dividing the indirect effect by the total effect.

### 2.8. Transcriptome Differential Analysis and GeneMANIA Analysis

To investigate whether the identified genes in the TWAS analysis were differentially expressed between EMT patients and healthy individuals, we performed transcriptome differential analysis. We retrieved a microarray dataset (GSE51981) from the Gene Expression Omnibus (GEO) database (https://www.ncbi.nlm.nih.gov/geo/, accessed on 10 August 2024), which encompasses the transcriptome profiles of endometrial samples of 77 EMT patients and 71 healthy individuals [35]. We employed the Wilcoxon test to evaluate whether significant differences existed in the expression of identified genes between EMT patients and healthy individuals. Furthermore, to visually represent our findings, we used R package “pheatmap” (V.1.0.12) and “ggpubr” (V.0.6.0).

GeneMANIA is a platform which integrates various genetic interactions, pathways, shared protein domains, co-localization, physical interactions, and co-expression datasets for target genes, as well as their gene–function relationships. We utilized this platform to further explore the biological functions of these identified genes [36].

### 2.9. Classification Hierarchy of Identified Gene Targets

The identified potential susceptibility genes were classified into four tiers according to four criteria: TWAS analyses, MR analyses, significant colocalization, and differential analyses. For TWAS analyses, if UTMOST, FUSION, and MAGMA all indicated that the gene is a significant susceptibility gene (FDR < 0.05), 1 point is assigned. For MR analyses, “Pass” represents 1 point, and “Not pass” represents 0 point. For significant colocalization, “Yes” represents 1 point, and “No” represents 0 point. For differential analyses, “Significant difference” represents 1 point, and “Not significant” represents 0 point. Finally, we calculated the total score for each protein, with a score of 4 being the tier 1 target, 3 being the tier 2 target, and 1 and 2 being the tier 3 target. For genes which did not present significant results in UTMOST, FUSION, and MAGMA, they were directly regarded as tier 4 targets.

## 3. Results

### 3.1. TWAS Analyses in Cross-Tissue and Single-Tissue

**Figure 2:**
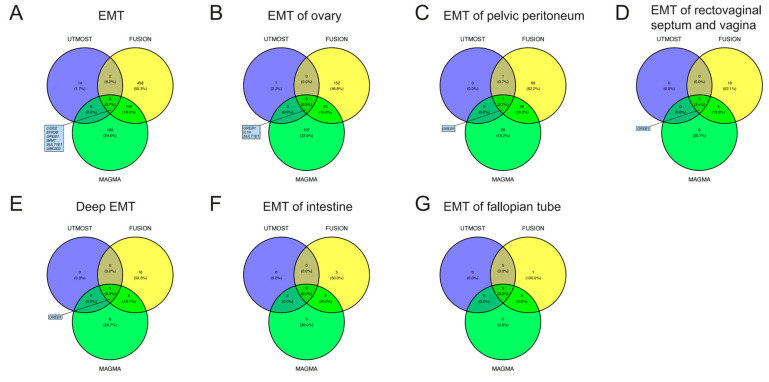
Venn diagrams. UTMOST, unified test for molecular signatures; FUSION, functional summary-based imputation; MAGMA, multi-marker analysis of genomic annotation; EMT, endometriosis.

For EMT, in cross-tissue TWAS (UTMOST) analyses, a total of 22 genes exhibited significant (FDR < 0.05) signal (Table S3a). Single-tissue TWAS (FUSION) identified 615 significant (FDR < 0.05) genes across 39 tissues, 8 of which had been identified via the UTMOST method (Table S3b). MAGMA gene-based analysis was also conducted, and it identified 354 significant (FDR < 0.05) genes (Table S3c). By integrating the results of UTMOST, FUSION, and MAGMA, six genes (CISD2, EFRB, GREB1, IMMT, SULT1E1, and UBE2D3) for EMT were identified which had demonstrated significant signal in all three analyses. Through similar procedures, we identified GREB1, IL1A, and SULT1E1 for EMT of the ovary, and we identified GREB1 for EMT of the pelvic peritoneum, EMT of the rectovaginal septum and vagina, and deep EMT. The Venn diagrams in Figure 2 illustrate the screening strategies and final results. The detailed results are presented in Tables S3–S9.

### 3.2. COJO Analyses

**Figure 3:**
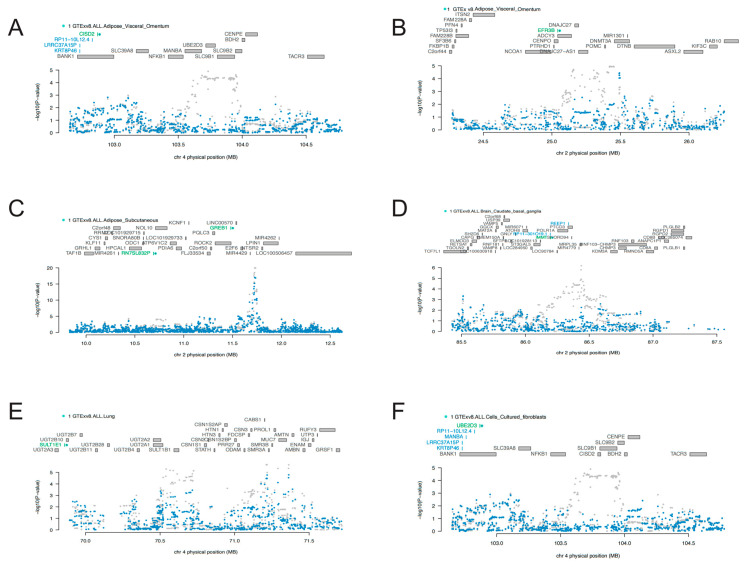
Regional association of TWAS hits: (**A**,**E**,**F**) showed chromosome 4 regional association plots; (**B**–**D**) showed chromosome 2 regional association plots. The top panel highlights all genes in the region. The marginally associated TWAS genes are shown in blue, and the jointly significant genes are shown in green. The bottom panel shows a regional Manhattan plot of GWAS data before (grey) and after (blue) conditioning on the predicted expression of the green genes.

Given that the seven identified candidate genes were predominantly located on chromosomes 2 and 4, we performed COJO analyses to mitigate the potential for false positive results resulting from LD. As is shown in Figure 3, marginally associated TWAS genes are shown in blue, and the jointly significant genes are shown in green. In “Adipose_Visceral_Omentum”, CISD2 stood as a jointly significant gene. However, conditional on the expression of CISD2, significant signals of KRT8P46, LRRC37A15P, and RP11-10L12.4 were notably reduced (Figure 3A). Similarly, after conditional analyses, a significant signal of EFR3B was found in “Adipose_Visceral_Omentum” (Figure 3B), GREB1 in “Adipose_Subcutaneous” (Figure 3C), IMMT in “Brain_Caudate_basal_ganglia” (Figure 3D), SULT1E1 in lung (Figure 3E), and UBE2D3 in “Cells_Cultured_fibroblasts” (Figure 3F). Results of the COJO analyses in other tissues are presented in Figures S1–S19. All these findings enhanced the robustness of the identified genes, which were not influenced by LD.

### 3.3. MR and Colocalization Analyses

To determine the causal associations between the identified genes and EMT, as well as the differences in transcriptional regulation among tissues, we employed eQTL data of the identified genes across different tissues (exhibiting significant results in FUSION analysis) and GWAS data of EMT to perform MR and SMR analyses. Due to the lack of valid (significant, independent, and strong) IVs for some genes (GREB1, SULT1E1, and IL1A) in the eQTL data of certain tissues, we ultimately investigated the causal associations between IMMT, EFR3B, and CISD, as well as UBE2D3 and EMT.

**Figure 4:**
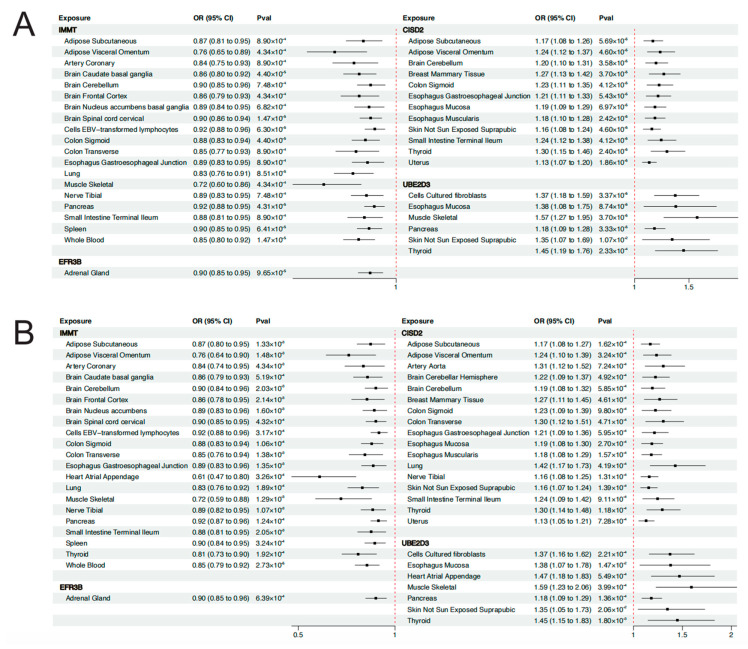
(**A**) Forest plot of MR analysis. (**B**) Forest plot of SMR analysis. OR, odds ratios; CI, confidence intervals; Pval, *p*-value.

**Figure 5:**
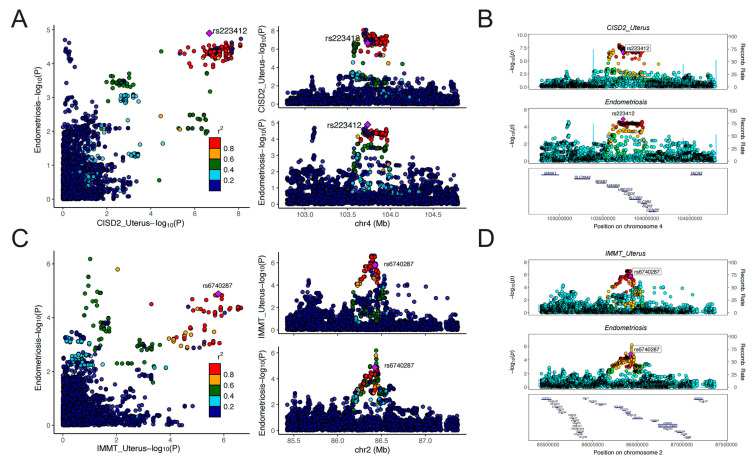
Regional association plots of colocalization analyses: (**A**,**B**) CISD2 in uterus and EMT; (**C**,**D**) IMMT in uterus and EMT. OR, odds ratio; CI, confidence interval; P_val, *p* value.

As was shown in Figure 4A, the expression level of IMMT in 19 tissues was causally associated with reduced risk of EMT. Expression level of EFR3B in the adrenal gland could reduce the risk of EMT (OR = 0.90, 95% CI: 0.85–0.95, *p* = 9.65 × 10^−5^). On the contrary, CISD2 and UBE2D3 served as risk genes for EMT. Genetically predicted levels of CISD2 in 12 tissues and UBE2D3 in 6 tissues presented causal associations with elevated risk of EMT. It is worth noting that the expression level of CISD2 in the uterus was associated with increased risk of EMT (OR = 1.13, 95% CI: 1.07–1.20, *p* = 1.86 × 10^−5^), which deserves further research. Detailed results of the MR analyses are presented in Table S10. Results of the Steiger filtering analyses provided assurance on the directionality of these causal associations (Table S10). The SMR analyses served to validate the findings of MR analyses (Figure 4B). Similarly, IMMT and EFR3B were identified as protective genes associated with EMT, whereas CISD2 and UBE2D3 were identified as risk genes for EMT. Through SMR analyses, the expression level of these genes across more tissues was found to be associated with EMT. For instance, the expression level of IMMT in 21 tissues, CISD2 in 17 tissues, and UBE2D3 in 7 tissues all demonstrated causal relationships with EMT risk. The HEIDI test found limited heterogeneity, suggesting that the associations were not caused by LD (Table S11). Detailed results of SMR analyses are presented in Table S11. The results of the colocalization analyses further supported our findings. Genetically predicted levels of CISD2 in 12 tissues, IMMT in 11 tissues, and UBE2D3 in the muscle–skeletal exhibited strong colocalization with EMT (PPH4 > 0.7). Genetically predicted levels of IL1A in the spleen presented colocalization with EMT of the ovary (PPH4 > 0.7). Figure 5 illustrates the regional association plots of colocalization analyses between CISD2 and IMMT in the uterus with EMT. Detailed significant results of the colocalization analyses are presented in Table S12.

### 3.4. Mediating Roles of Modifiable Risk Factors in the Association Between Identified Genes Across Tissues and EMT

**Figure 6:**
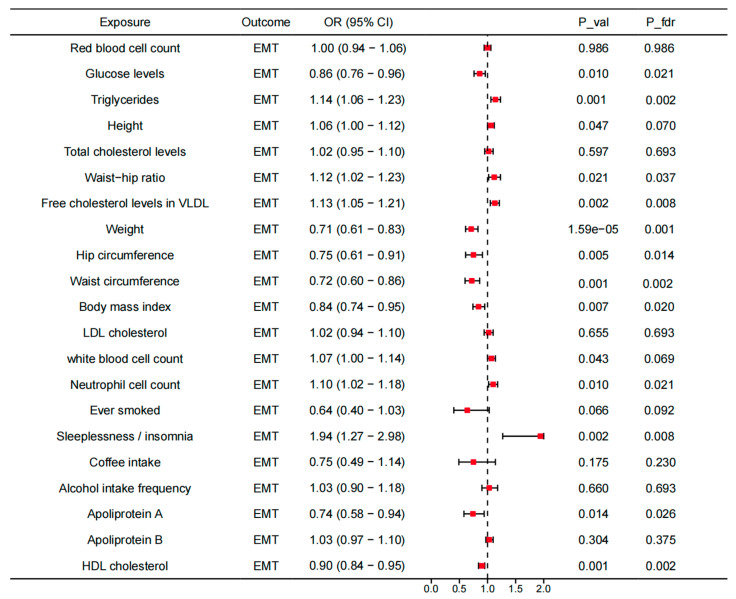
Causal associations between modifiable risk factors and the risk of EMT. OR, odds ratio; CI, confidence interval; P_val, *p*-value; P_fdr, *p*-value corrected via false discovery rate (FDR) method.

**Figure 7:**
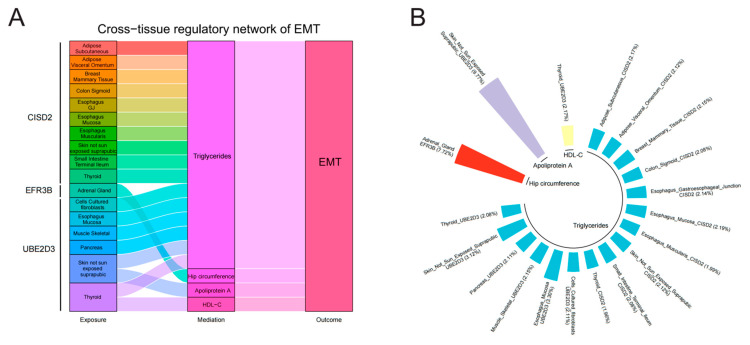
Two-step network mediation analysis connecting genetic proxies for identified genes across various tissues to EMT through potential mediators: (**A**) overview of pathways linking identified genes across various tissues to EMT; (**B**) proportion of association between genetic proxies for identified genes across various tissues and EMT mediated by potential mediators. The bar chart is labeled as “Exposure (mediating proportion)”.

To investigate the underlying mechanisms, we investigated the associations between the identified genes across the tissues and the modifiable risk factors, as well as the associations between the modifiable risk factors and EMT (Supplemental Tables S13 and S14). For 21 modifiable risk factors, 14 present causal associations with EMT (*p* < 0.05). After FDR correction, the causal associations between glucose levels, TG, waist–hip ratio, VLDL-C, weight, hip circumference, waist circumference, BMI, LDL-C, neutrophil count, insomnia, apolipoprotein A, and HDL-C and EMT were still significant (Figure 6). All the analyses have passed the Steiger filtering test (Supplemental Tables S13 and S14). Although heterogeneity was detected in some associations (Table S15), no horizontal pleiotropy was observed (Table S16). Subsequently, we performed two-sample network MR analyses and established a mediating network connecting identified genes across tissues and EMT via modifiable risk factors. Finally, we identified a total of 19 significant (*p* < 0.05) pathways with directional consistency (Figure 7A). The mediation proportion ranged from 1.96% to 9.77% (Figure 7B). The detailed results of mediation analysis (*p*-values, indirect effect, and their 95% CI) are presented in Supplemental Table S17. In summary, genetically predicted expression levels of CISD across different tissues increased the risk of EMT by up-regulating the levels of TG. EFR3B in the adrenal gland could reduce the risk of EMT by increasing hip circumference. UBE2D3 elevated EMT risk by regulating the levels of TG, apolipoprotein A, and HDL-C.

### 3.5. Further Bioinformatics Analyses

Figures S20–S26 illustrates the potential gene interaction networks constructed with the seven identified genes as the core. Function enrichment analyses revealed that CISD2-related genes played roles in mitochondrial transport and the positive regulation of the apoptotic signaling pathway (Table S18). The most significant functional pathways enriched in IMMT-related gene networks were integral components of mitochondrial inner membrane and inner mitochondrial membrane protein complex (Table S18). In the case of SULT1E1-related gene networks, the most significant functional pathways were purine ribonucleoside bisphosphate metabolic process and sulfotransferase activity (Table S18). For UBE2D3-related gene networks, the key functional pathways enriched were ubiquitin–protein transferase activity and the MyD88-independent toll-like receptor signaling pathway (Table S18). Additionally, the most significant functional pathways enriched in IL1A-related gene networks were the cellular response to interleukin-1 and the positive regulation of DNA-binding transcription factor activity (Table S18). However, for GREB1 and EFR3B, no significant functional pathways were enriched.

To further investigate whether the identified genes in TWAS analysis were differentially expressed between EMT patients and healthy individuals, we performed transcriptome differential analysis (Figures S27–S34). The heatmap was illustrated in Figure S27. Finally, the expression levels of EFR3B (Figure S29, *p* = 0.00023), GREB1 (Figure S30, *p* = 0.017), and IMMT (Figure S32, *p* = 0.0011) were significantly lower in the EMT group. In our previous steps, we had confirmed IMMT and EFR3B as protective genes which could reduce the risk of EMT via MR and SMR analyses. Here, lower expression of the two genes further validated these findings.

### 3.6. Classification Hierarchy of Identified Gene Targets

Finally, we calculated the scores of the identified targets and classified them into four tiers according to the aforementioned criteria. Detailed information is presented in Table S19. IMMT was regarded as a tier 1 target; CISD2, EFR3B, and UBE2D3 as tier 2 targets; GREB1, SULT1E1, and IL1A as tier 3 targets; and other identified targets (Tables S3–S9) as tier 4 targets.

## 4. Discussion

Leveraging the latest EMT GWAS and GTEx v8 eQTL data, we conducted a comprehensive assessment of the associations between genetically predicted levels of gene expression and EMT. Through cross-tissue TWAS, single-tissue TWAS, and MAGMA analyses, we identified a total of seven promising gene targets (CISD2, EFR3B, GREB1, IMMT, SULT1E1, UBE2D3, and IL1A) for EMT. MR and SMR analyses further elucidated the causal associations between thr expression level of genes across diverse tissues and EMT risk, shedding light on the tissue-specific transcriptional regulatory patterns of EMT. As for specific mechanisms, CISD2, EFR3B, and UBE2D3 across tissues could regulate the levels of blood lipids (TG, apolipoprotein A, and HDL-C) and hip circumference so as to influence the risk of EMT. Colocalization analyses and bioinformatics analyses bolstered the reliability of our findings and enhanced our understanding of the potential functions of these susceptibility genes.

Currently, the integration of quantitative trait loci (QTL) data and GWAS data in multi-omics association analyses is widely employed to identify potential therapeutic targets for diseases. Tao et al. leveraged the protein QTL data of 2923 plasma proteins form the UK Biobank Pharmaceutical Proteomics Project (UKB-PPP) to scrutinize therapeutic targets exhibiting causal associations with EMT through MR and colocalization analyses [37]. Likewise, Zeng et al. performed MR analyses using eQTL data and the GWAS data of EMT [38]. Another study delved into DNA methylation, utilizing methylation QTL data to identify potential loci for EMT [39]. Consistent with our findings, many methylation loci were prominently located in proximity to GREB1, underscoring its significance as a target for EMT. Nevertheless, these investigations only focused on specific tissues (such as uterus and whole blood) for identifying potential therapeutic targets. Given the tissue dependence and resemblance of the transcriptional regulation in the body, concentrating solely on a few tissues has significant limitations. Conducting cross-tissue association analyses may potentially yield more profound and insightful findings. At present, only one study conducted by Mortlock et al. employed RNA sequencing (RNA-seq) data and GTEx dataset for cross-tissue analysis to screen potential risk loci for EMT [40]. Unveiling 327 novel eQTLs from RNA-seq data, the study proceeded to conduct correlation analysis, leveraging eQTL data across diverse tissues in the GTEx dataset. Additionally, the authors also conducted TWAS analysis using the transcriptome-integrated genetic association resource (TIGAR) tool and validated causal associations using SMR analysis. However, this study has significant limitations. First, correlation analysis between eQTLs in different tissues cannot clarify the direct effect of eQTLs across diverse tissues on EMT, and the level of evidence is relatively weak. Furthermore, the results obtained from this study are not convincing. In SMR analyses, a notable portion of results failed the HEIDI test (*p* < 0.05), indicating that the effects of these genes are likely caused by LD. Therefore, the reliability of their findings still needs further discussion [40]. Studies have indicated that cross-tissue TWAS analysis exhibited greater statistical efficacy in identifying genes associated with complex traits, thereby enhancing our ability to detect missing associations when using single-tissue TWAS analysis [12,41]. In our study, eQTL data across different tissues from GTEx v8 were utilized to conduct both cross-tissue TWAS (UTMOST) analyses and single-tissue TWAS (FUSION) analyses. MAGMA analyses were conducted to validate our findings. For causal associations, MR and SMR analyses were performed, and limited heterogeneity was detected via the HEIDI test (*p* > 0.05). Colocalization and bioinformatics analyses further enriched and supported our findings. Ultimately, we identified seven novel gene targets for EMT. Among these targets, CISD2, EFR3B, UBE2D3, and IMMT have not been reported previously, and we further delved into their transcriptional regulatory patterns associated with EMT. As for specific mechanisms, CISD2, EFR3B, and UBE2D3 across tissues could regulate the levels of blood lipids (TG, apoliprotein A and HDL-C) and hip circumference so as to influence the risk of EMT. These findings have important clinical significance. On the one hand, the expression levels of these genes across various tissues deserve further in-depth exploration; on the other hand, monitoring blood lipids and hip circumference is helpful for early diagnosis and prevention of EMT. In the future, the regulation mechanism of lipid levels by the identified genes could provide new insights into the pathophysiological mechanism of EMT. If necessary, relevant clinical trials could be conducted to promote the development of new drugs and achieve clinical translation.

GREB1, SULT1E1, and IL1A have been previously reported to be associated with EMT risk and were identified as susceptibility genes for both EMT and EMT of the ovary in our study. Growth regulating estrogen receptor binding 1 (GREB1) is an estrogen-responsive gene and is classified as an early response gene within the estrogen receptor-regulated pathway. Previous studies have demonstrated that GREB1 played an important role in hormone-responsive tissues and cancer [42,43]. For EMT, GREB1 was also found to play a significant role. Chadchan et al. reported that GREB1 bound to progesterone receptors to control the progesterone responses in the uterine stroma, thereby affecting endometrial receptivity and decidualization [44]. Furthermore, another two studies have shown that methylation loci prominently located in proximity to GREB1 are closely associated with EMT risk [39,45]. In conclusion, significant genetic associations have been established between GREB1 and the risk of EMT [46,47,48]. In our study, GREB1 was identified as a susceptibility gene not only for EMT but also for four EMT subtypes. Single-tissue TWAS (FUSION) analyses revealed that the expression levels of GREB1 in “Brain_Substantia_nigra”, “Cells_Cultured_fibroblasts”, and “Minor_Salivary_Gland” were intricately linked to EMT risk, thereby providing novel insights into the transcriptional regulatory mechanisms of GREB1 across tissues. Nevertheless, due to the limitation of eQTL data, we failed to obtain IVs for GREB1 and to conduct MR analyses to explore the causal associations. Future investigations should concentrate on elucidating the effect of expression of GREB1 in specific tissue on EMT risk, with the aim of uncovering new biological mechanisms. Sulfotransferase family 1E member 1 (SULT1E1) encodes a protein that transfers a sulfo moiety to and from estrone, which may control levels of estrogen receptors [49]. Interleukin 1 alpha (IL1A) is a pleiotropic cytokine involved in various immune responses, inflammatory processes, and hematopoiesis [50]. In our study, SULT1E1 and IL1A were identified as potential targets for EMT of the ovary, which have been seldom reported. We anticipate that these two genes will become specific therapeutic targets for EMT of the ovary.

CISD2, UBE2D3, IMMT, and EFR3B were identified as novel susceptibility genes which have never been reported in previous studies. We also employed MR and SMR analyses to investigate the causal relationship between their expression and EMT. To be specific, CISD and UBE2D3 were regarded as risk genes for EMT, while IMMT and EFR3B were regarded as protective genes for EMT. Furthermore, the expression level of CISD2 in 17 tissues, UBE2D3 in 7 tissues, IMMT in 21 tissues, and EFR3B in the adrenal gland demonstrated causal relationships with EMT risk. As for mechanisms, CISD2 across tissues increased the level of TG, which, in turn, increased the risk of EMT. EFR3B in the adrenal gland was positively associated with hip circumference and could reduce the risk of EMT. UBE2D3 influenced the risk of EMT by regulating the levels of TG, apolipoprotein A, and HDL-C. CDGSH iron sulfur domain 2 (CISD2) is a zinc finger protein located in the endoplasmic reticulum (ER), which plays a crucial in regulating cytoplasmic calcium homeostasis, ER integrity, and mitochondrial function [51]. At present, studies on CISD2 mainly focus on age-related diseases and cancer. CISD2 have been proven to have anti-aging properties, while for tumors, it could act as a double-edged sword [52,53,54]. Zhou et al. reported that CISD2 was involved in the mitochondrial transport of LDL, but there is currently no research on the association between CISD2 and TG. We have speculated that CISD2 might be closely related to lipid transport and could regulate the levels of various lipids in the body so as to increase the risk of EMT [55]. Ubiquitin conjugating enzyme E2 D3 (UBE2D3), a member of the E2 ubiquitin-conjugating enzyme family, functions in the ubiquitination of the tumor-suppressor protein p53, which is induced by an E3 ubiquitin–protein ligase [56]. Pan et al. reported that UBE2D3 could promote the ubiquitination of SHP2, thereby activating the STAT3 pathway and promoting glioma proliferation [57]. Wang et al. demonstrated that UBE2D3 promoted p62 ubiquitination to aggravate the impairment of autophagic flux, which contributed to myocardial ischemia-reperfusion injury [58]. Given the close association between UBE2D3 and ubiquitination, and our findings indicating that UBE2D3 can increase the risk of EMT by regulating lipid levels, we speculate that UBE2D3 is likely to be associated with the degradation of some lipid transport proteins, thereby affecting lipid transport and increasing the risk of EMT. Inner-membrane mitochondrial protein (IMMT) is located in the mitochondrial inner membrane, which enables RNA-binding activity [59]. Hiyoshi et al. reported that the expression level of IMMT was an independent indicator of poor survival prognosis in patients with lung adenocarcinoma [59]. In Liu et al.’s study, IMMT was proven to promote the proliferation of breast cancer cells by regulating mitochondrial metabolism [60]. EFR3 homolog B (EFR3B), located in actin cytoskeleton, cytosol, and the plasma membrane, plays an important role in the phosphatidylinositol phosphate biosynthetic process and protein localization to the plasma membrane [61]. Wei et al. have demonstrated that EFR3B plays a significant role in social novelty by modulating the excitability of CA2 pyramidal neurons [62]. Subsequent investigations can be built upon our findings to delve into the underlying mechanisms by which these genes affect EMT risk across tissues in order to construct a more comprehensive genetic architecture for EMT.

This study has several strengths. First, this is the first cross-tissue TWAS analysis for EMT, through which we identified seven reliable gene targets for EMT. Our findings provided fresh insights into the tissue-specific transcriptional regulatory patterns of EMT. Second, our research methodology is robust and diverse. In the TWAS analysis stage, we employed both cross-tissue (UTMOST) and single-tissue (FUSION) TWAS analyses, complimented by MAGMA analysis to validate our findings. Subsequently, MR and SMR analyses were conducted to delve into the causal associations between gene expression levels across tissues and EMT. Moreover, two-sample network MR analyses revealed the potential mechanisms of the causal associations between identified genes across tissues and EMT. Finally, colocalization analyses and bioinformatics analyses further enhanced the reliability of the study.

However, some limitations cannot be ignored. First, data utilized in this study were exclusively from European individuals. Hence, further research is needed to validate the applicability of our findings to other racial and ethnic groups. At present, most GWAS and TWAS data are form Europeans. However, once the data of other populations are available, we will conduct immediate analyses. Furthermore, we also consider recruiting Chinese people for sequencing and carry out relevant analysis for East Asian people. After obtaining results from different ethnic groups, we consider conducting meta-analysis to further validate the consistency of the results. Second, the data of some tissues in GTEx v8 only included the eQTL of limited genes. Consequently, the causal associations between these genes (such as GREB1, SULT1E1, and IL1A) and EMT cannot be evaluated using MR analysis. Third, in this study, we used the GWAS data of many EMT subtypes to identify the susceptibility genes for EMT occurring in specific regions. Unfortunately, most of the final results were targeted at EMT, and we were unable to identify specific gene targets for EMT of the pelvic peritoneum, rectovaginal septum, vagina, and other sites. In the future, we will need larger-scale GWAS data to draw reliable conclusions. At last, due to funding constraints and ethical factors, we are currently unable to conduct basic research and clinical trials to prove our findings.

## 5. Conclusions

In summary, we identified seven novel susceptibility genes whose genetically predicted expression level was associated with EMT risk, providing fresh perspectives on the underlying genetic framework of EMT. MR analyses, colocalization analyses, and bioinformatics analyses further bolstered the robustness of our findings. Nevertheless, additional basic and functional investigations are still needed to elucidate the underlying biological mechanisms associated with these potential genes in different tissues.

## Data Availability

All data generated or analyzed during this study are included in this article and its Supplementary Materials. Additional materials can be obtained from the corresponding author upon reasonable request.

## Supplementary Materials

The following supporting information can be downloaded at https://www.mdpi.com/article/10.3390/biology13110871/s1: Table S1. The sources for all statistical summary datasets used in this study; Table S2. Instrumental variables for modifiable risk factors; Table S3. The results of TWAS analyses for EMT; Table S4. The results of TWAS analyses for EMT of ovary; Table S5. The results of TWAS analyses for EMT of pelvic peritoneum; Table S6. The results of TWAS analyses for EMT of rectovaginal septum and vagina; Table S7. The results of TWAS analyses for deep EMT; Table S8. The results of TWAS analyses for EMT of intestine; Table S9. The results of TWAS analyses for EMT of fallopian tube; Table S10. Detailed results of MR analyses; Table S11. Detailed results of SMR analyses; Table S12. Detailed results of colocalization results of eQTLs with EMT-associated SNPs; Table S13. MR results between modifiable risk factors and EMT; Table S14. MR results between identified genes across tissues and modifiable risk factors; Table S15. Results of Cochran’s Q test; Table S16. Results of MR Egger intercept test; Table S17. Estimated mediation of blood proteins in the associations between anthropometric measurements, lifestyle factors, blood lipids, and EMT; Table S18. Functional pathway analysis; Table S19. Distinct target groups of identified proteins; Figures S1–S19. Regional association of TWAS hits; Figures S20–S26, GeneMANIA gene network; Figure S27. The heatmap of identified genes between EMT and control groups. Red and blue grids represent up- and down-regulated genes, respectively. CT, Control; EMT, Endometriosis; Figures S28–S34 The expression differences of 7 identified genes between EMT group and CT group.
