# Supplementary material for: Cross-Tissue Regulatory Network Analyses Reveal Novel Susceptibility Genes and Potential Mechanisms for Endometriosis
Source: Biology (Basel). 2024 Oct 26;13(11):871. doi: 10.3390/biology13110871 (PMC11591882; doi:10.3390/biology13110871)
Supplement: Supplementary file 1 [file biology-13-00871-s001.zip › Supplementary_Figures.pdf]

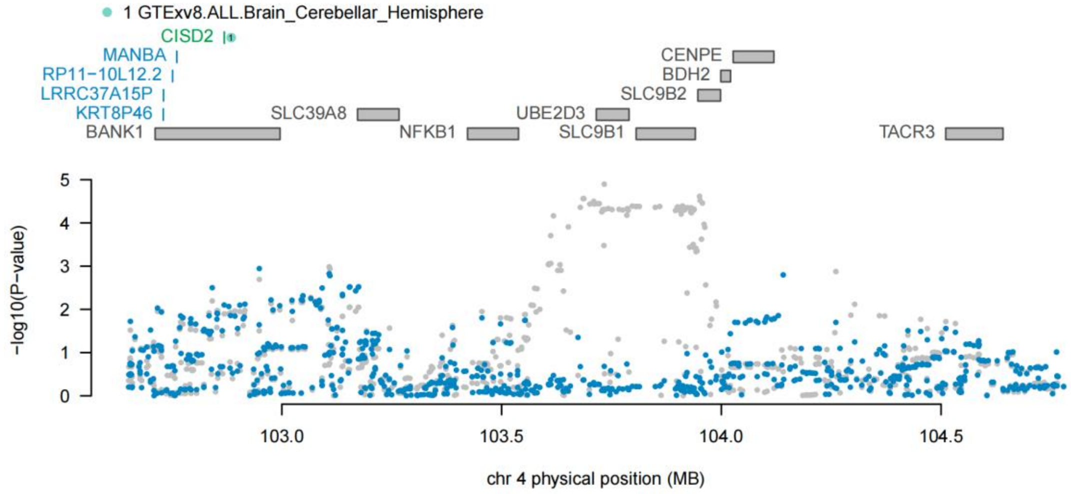

Figure S1. Regional association of TWAS hits. The top panel highlights all genes in the region. The marginally associated TWAS genes are shown in blue, and the jointly significant genes are shown in green. The bottom panel shows a regional Manhattan plot of GWAS data before (grey) and after (blue) conditioning on the predicted expression of the green genes.

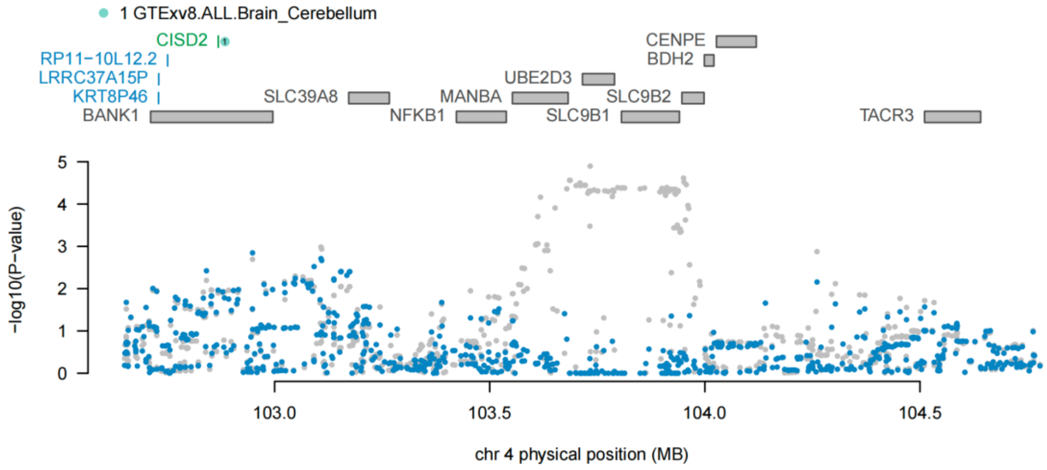

Figure S2. Regional association of TWAS hits. The top panel highlights all genes in the region. The marginally associated TWAS genes are shown in blue, and the jointly significant genes are shown in green. The bottom panel shows a regional Manhattan plot of GWAS data before (grey) and after (blue) conditioning on the predicted expression of the green genes.

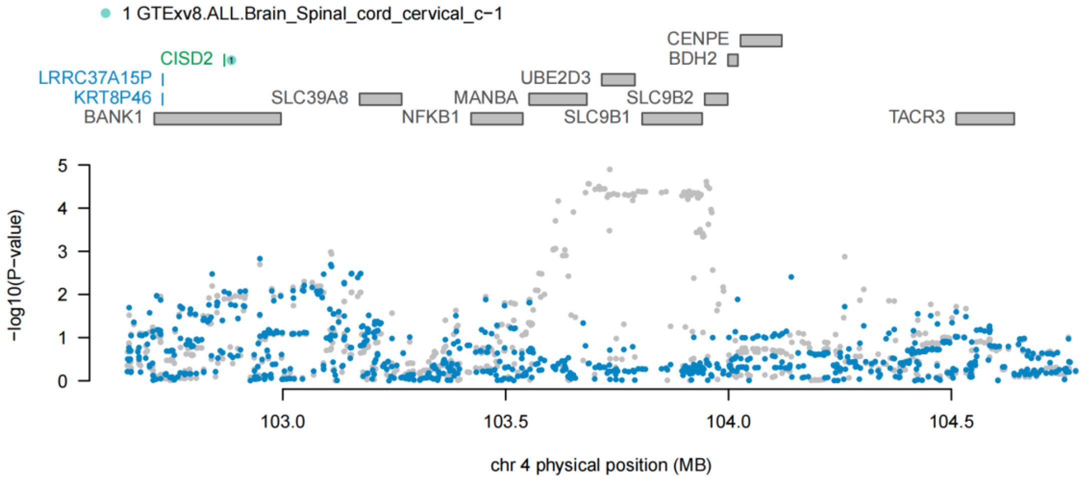

Figure S3. Regional association of TWAS hits. The top panel highlights all genes in the region. The marginally associated TWAS genes are shown in blue, and the jointly significant genes are shown in green. The bottom panel shows a regional Manhattan plot of GWAS data before (grey) and after (blue) conditioning on the predicted expression of the green genes.

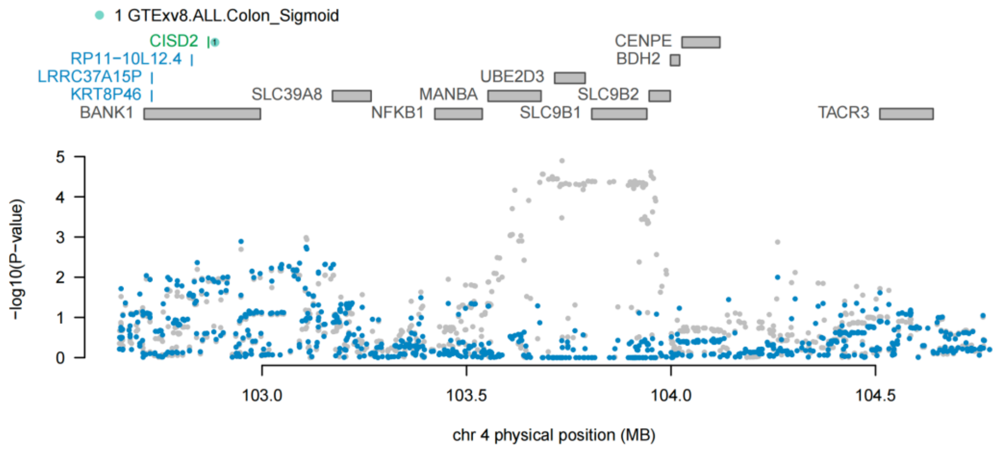

Figure S4. Regional association of TWAS hits. The top panel highlights all genes in the region. The marginally associated TWAS genes are shown in blue, and the jointly significant genes are shown in green. The bottom panel shows a regional Manhattan plot of GWAS data before (grey) and after (blue) conditioning on the predicted expression of the green genes.

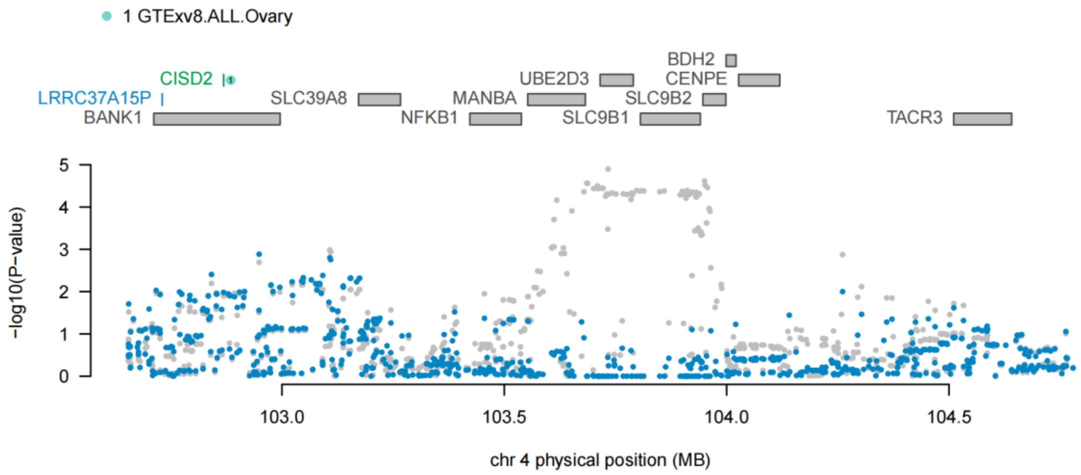

Figure S5. Regional association of TWAS hits. The top panel highlights all genes in the region. The marginally associated TWAS genes are shown in blue, and the jointly significant genes are shown in green. The bottom panel shows a regional Manhattan plot of GWAS data before (grey) and after (blue) conditioning on the predicted expression of the green genes.

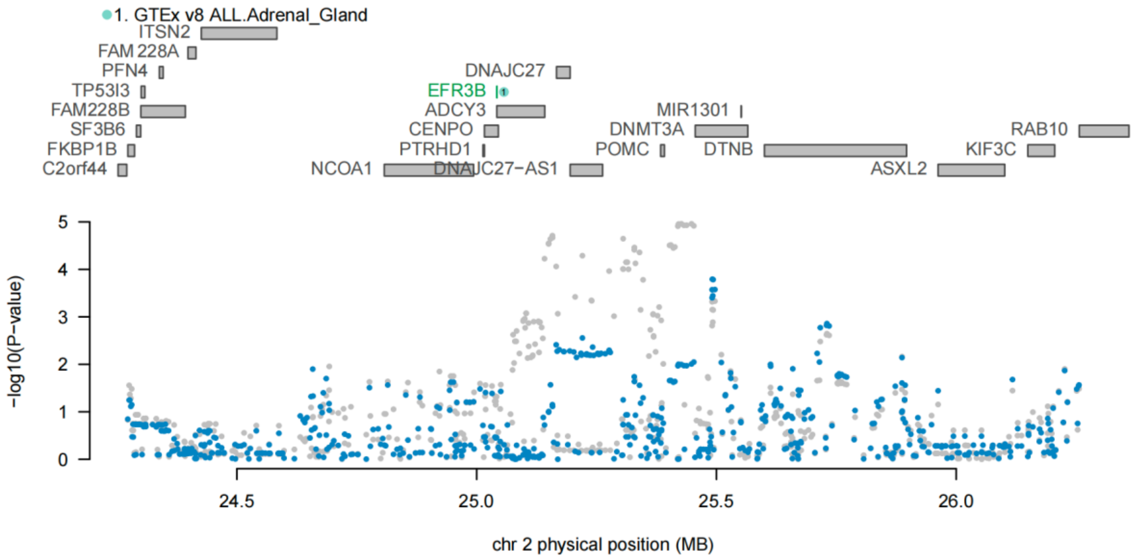

Figure S6. Regional association of TWAS hits. The top panel highlights all genes in the region. The marginally associated TWAS genes are shown in blue, and the jointly significant genes are shown in green. The bottom panel shows a regional

Manhattan plot of GWAS data before (grey) and after (blue) conditioning on the predicted expression of the green genes.

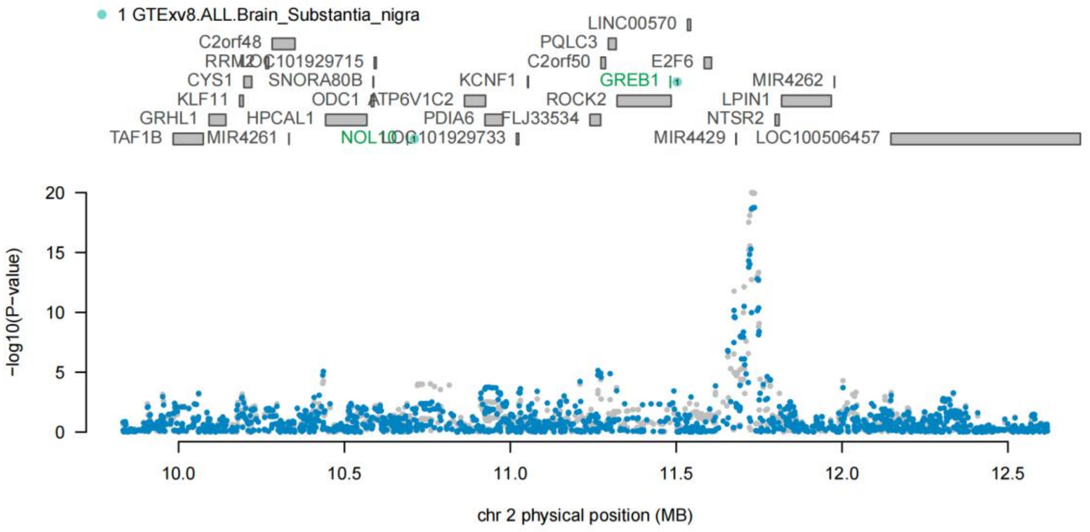

Figure S7. Regional association of TWAS hits. The top panel highlights all genes in the region. The marginally associated TWAS genes are shown in blue, and the jointly significant genes are shown in green. The bottom panel shows a regional Manhattan plot of GWAS data before (grey) and after (blue) conditioning on the predicted expression of the green genes.

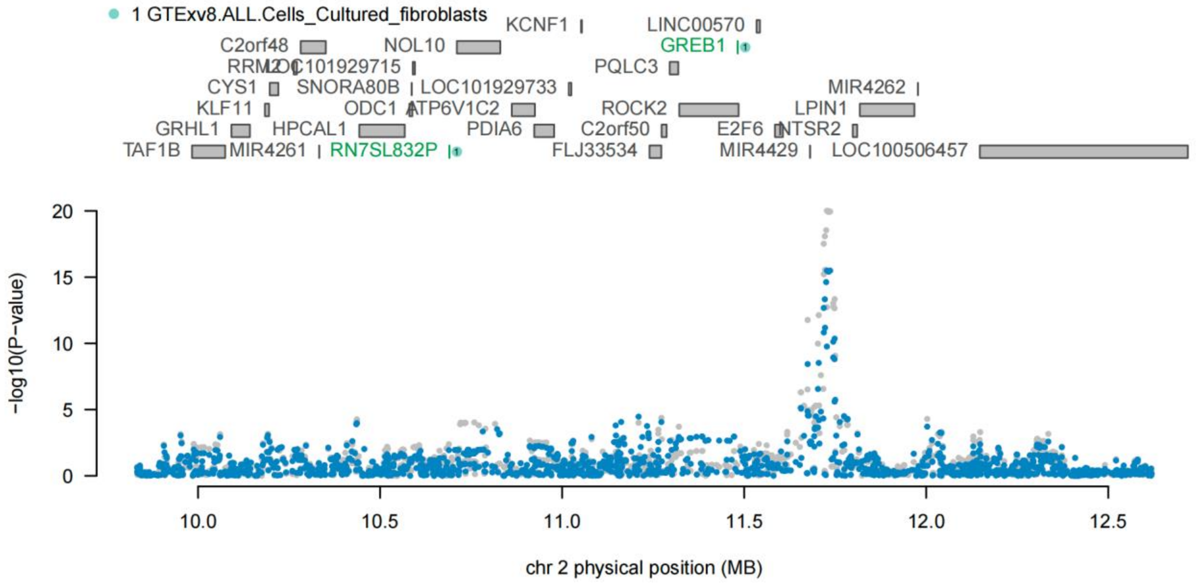

Figure S8. Regional association of TWAS hits. The top panel highlights all genes in the region. The marginally associated TWAS genes are shown in blue, and the jointly significant genes are shown in green. The bottom panel shows a regional Manhattan plot of GWAS data before (grey) and after (blue) conditioning on the predicted expression of the green genes.

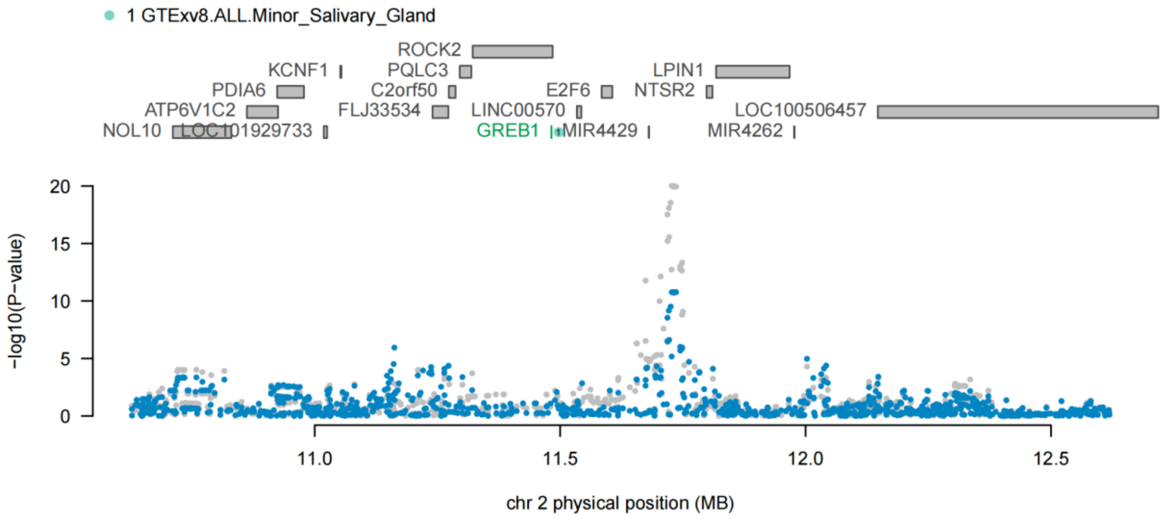

Figure S9. Regional association of TWAS hits. The top panel highlights all genes in the region. The marginally associated TWAS genes are shown in blue, and the jointly significant genes are shown in green. The bottom panel shows a regional Manhattan plot of GWAS data before (grey) and after (blue) conditioning on the predicted expression of the green genes.

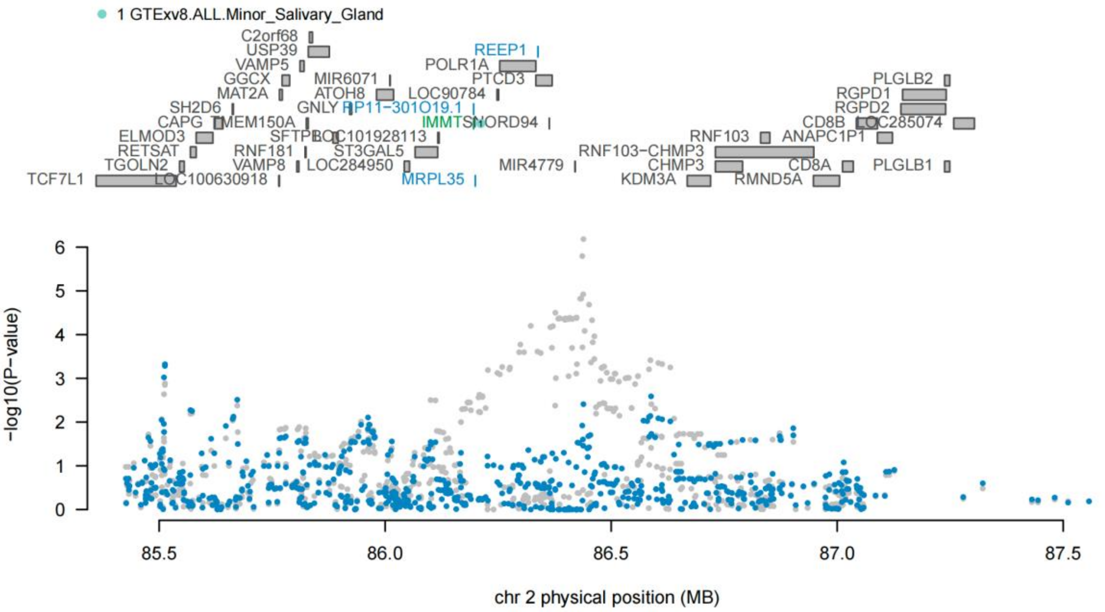

Figure S10. Regional association of TWAS hits. The top panel highlights all genes in the region. The marginally associated TWAS genes are shown in blue, and the jointly significant genes are shown in green. The bottom panel shows a regional Manhattan plot of GWAS data before (grey) and after (blue) conditioning on the predicted expression of the green genes.

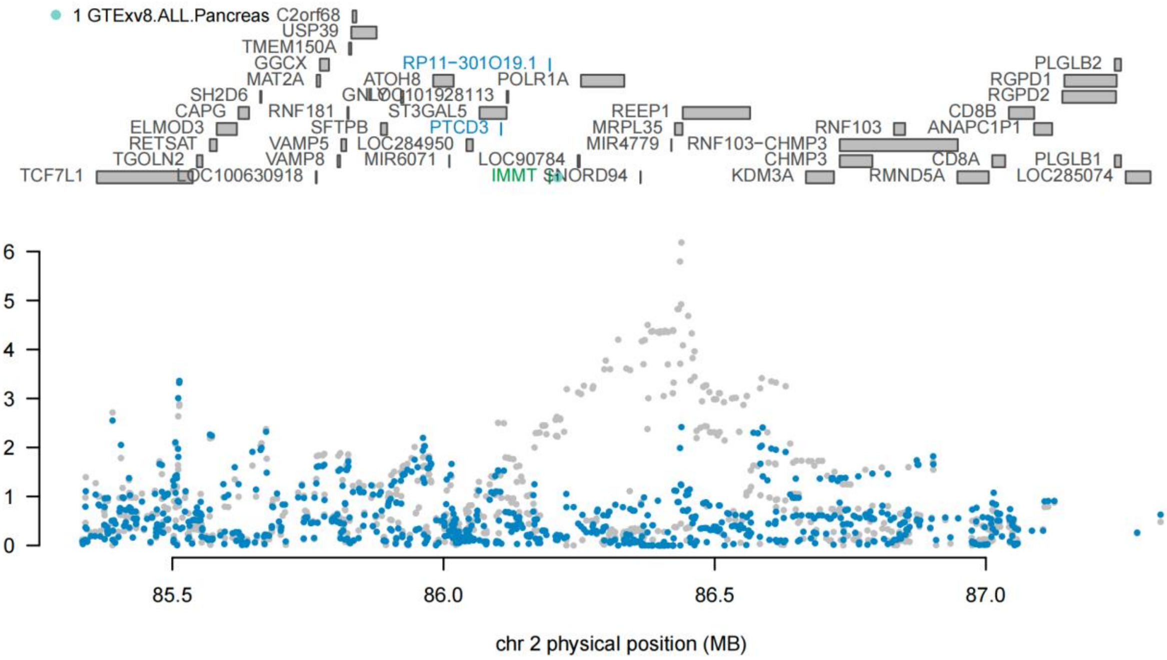

Figure S11. Regional association of TWAS hits. The top panel highlights all genes in the region. The marginally associated TWAS genes are shown in blue, and the jointly significant genes are shown in green. The bottom panel shows a regional Manhattan plot of GWAS data before (grey) and after (blue) conditioning on the predicted expression of the green genes.

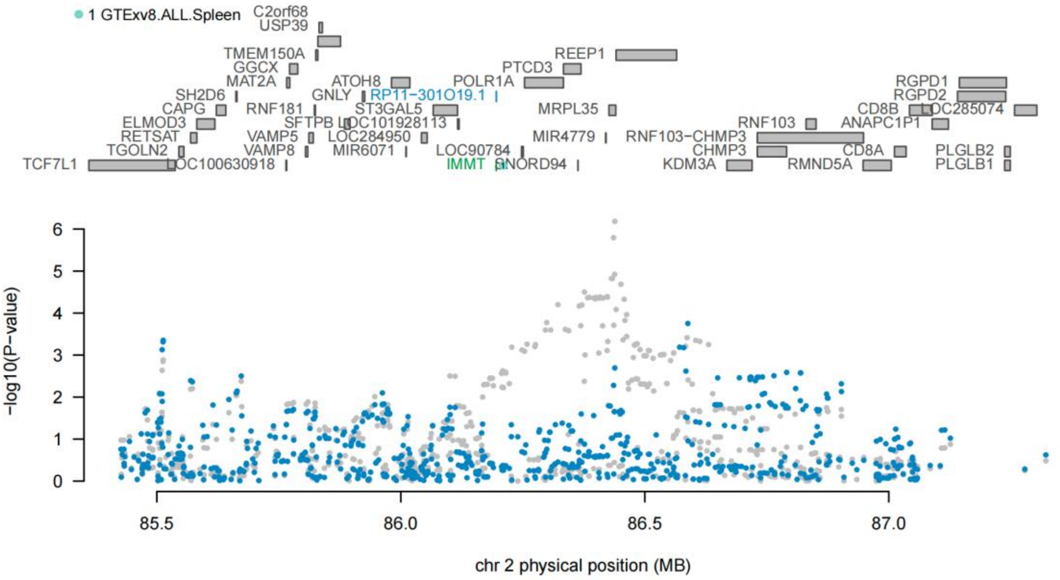

Figure S12. Regional association of TWAS hits. The top panel highlights all genes in the region. The marginally associated TWAS genes are shown in blue, and the jointly significant genes are shown in green. The bottom panel shows a regional Manhattan plot of GWAS data before (grey) and after (blue) conditioning on the predicted expression of the green genes.

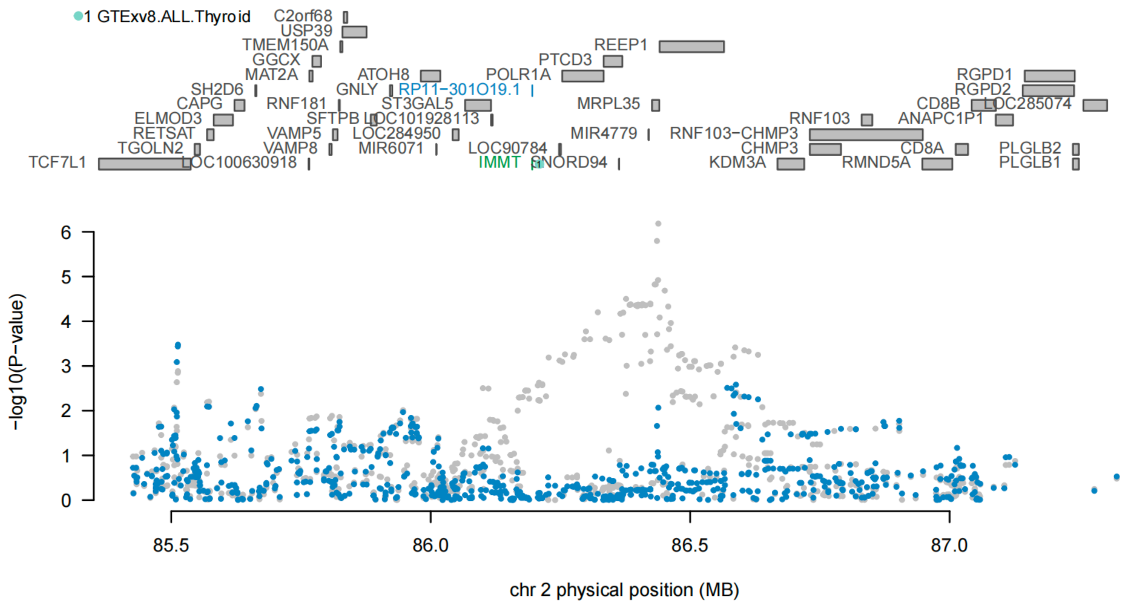

Figure S13. Regional association of TWAS hits. The top panel highlights all genes in the region. The marginally associated TWAS genes are shown in blue, and the jointly significant genes are shown in green. The bottom panel shows a regional Manhattan plot of GWAS data before (grey) and after (blue) conditioning on the predicted expression of the green genes.

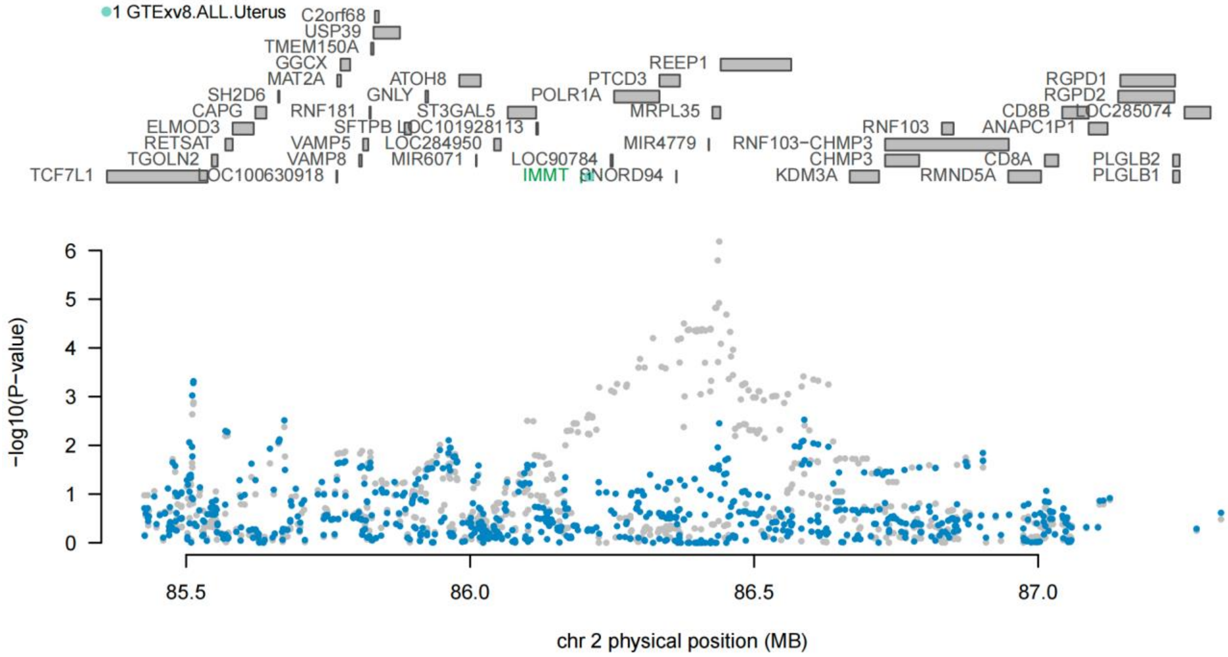

Figure S14. Regional association of TWAS hits. The top panel highlights all genes in the region. The marginally associated TWAS genes are shown in blue, and the jointly significant genes are shown in green. The bottom panel shows a regional Manhattan plot of GWAS data before (grey) and after (blue) conditioning on the predicted expression of the green genes.

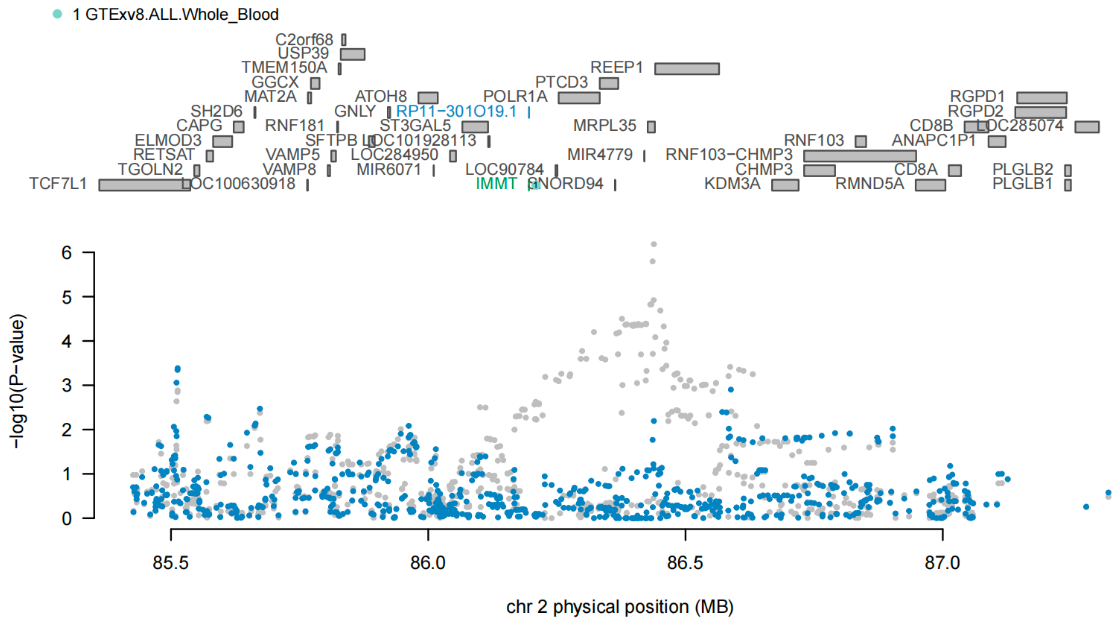

Figure S15. Regional association of TWAS hits. The top panel highlights all genes in the region. The marginally associated TWAS genes are shown in blue, and the jointly significant genes are shown in green. The bottom panel shows a regional Manhattan plot of GWAS data before (grey) and after (blue) conditioning on the predicted expression of the green genes.

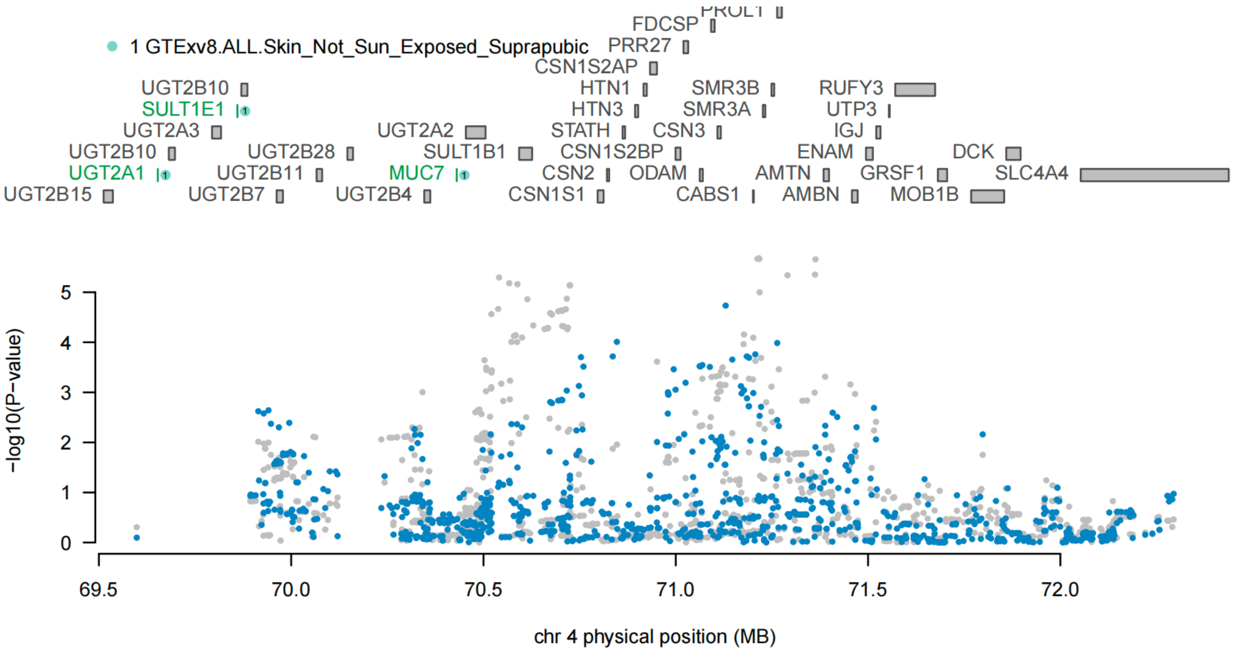

Figure S16. Regional association of TWAS hits. The top panel highlights all genes in the region. The marginally associated TWAS genes are shown in blue, and the jointly significant genes are shown in green. The bottom panel shows a regional Manhattan plot of GWAS data before (grey) and after (blue) conditioning on the predicted expression of the green genes.

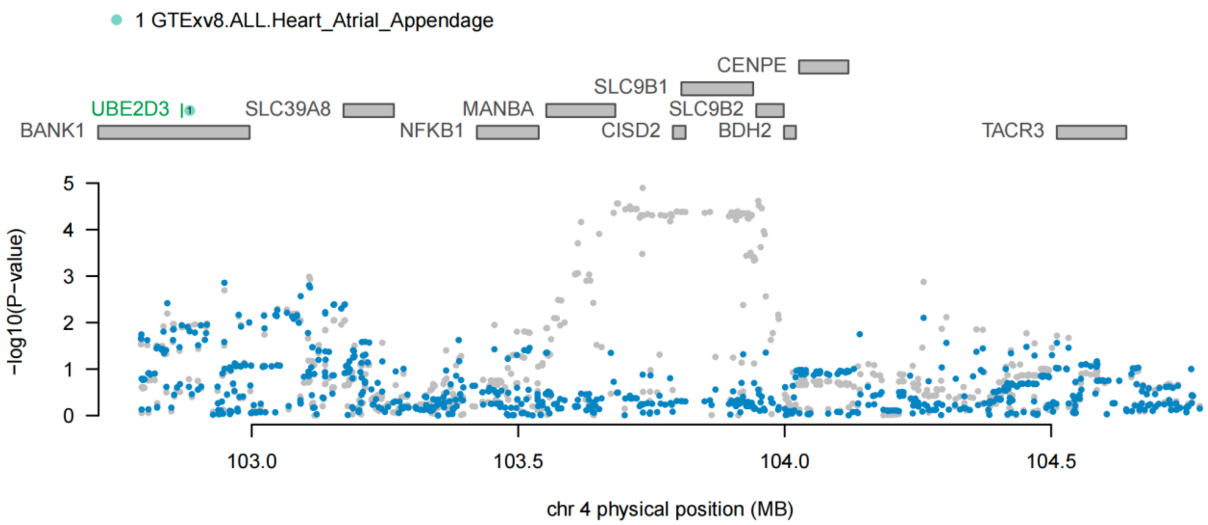

Figure S17. Regional association of TWAS hits. The top panel highlights all genes in the region. The marginally associated TWAS genes are shown in blue, and the jointly significant genes are shown in green. The bottom panel shows a regional Manhattan plot of GWAS data before (grey) and after (blue) conditioning on the predicted expression of the green genes.

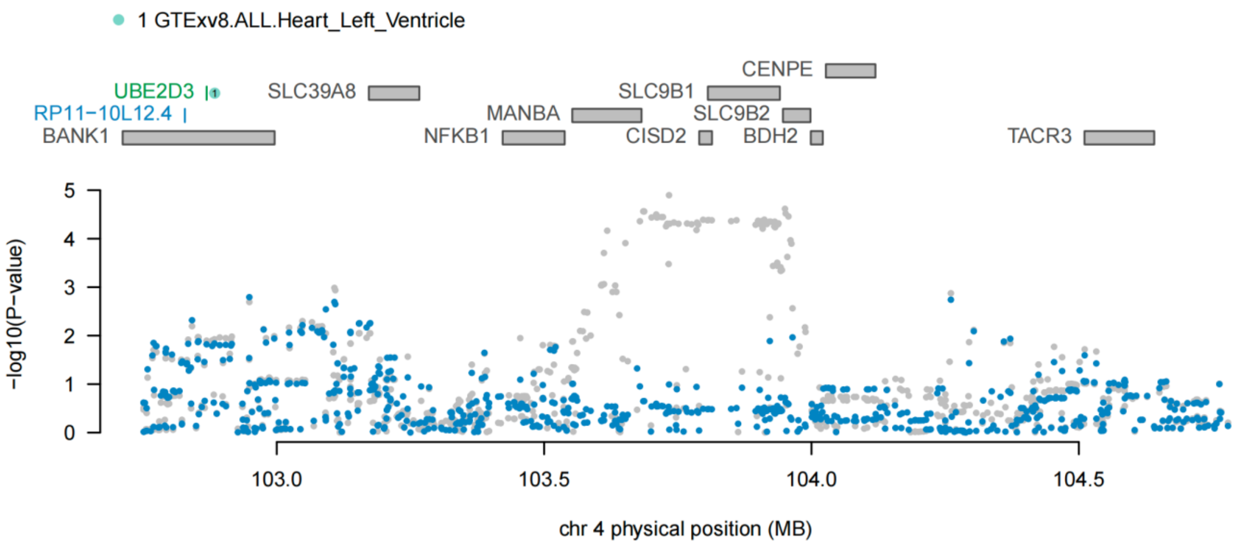

Figure S18. Regional association of TWAS hits. The top panel highlights all genes in the region. The marginally associated TWAS genes are shown in blue, and the jointly significant genes are shown in green. The bottom panel shows a regional Manhattan plot of GWAS data before (grey) and after (blue) conditioning on the predicted expression of the green genes.

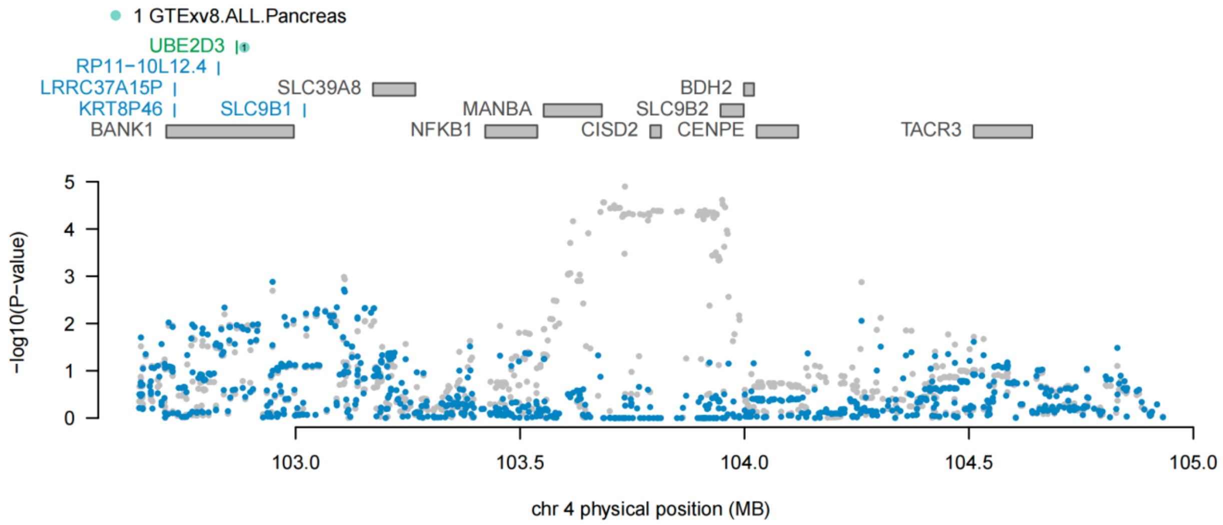

Figure S19. Regional association of TWAS hits. The top panel highlights all genes in the region. The marginally associated TWAS genes are shown in blue, and the jointly significant genes are shown in green. The bottom panel shows a regional Manhattan plot of GWAS data before (grey) and after (blue) conditioning on the predicted expression of the green genes.

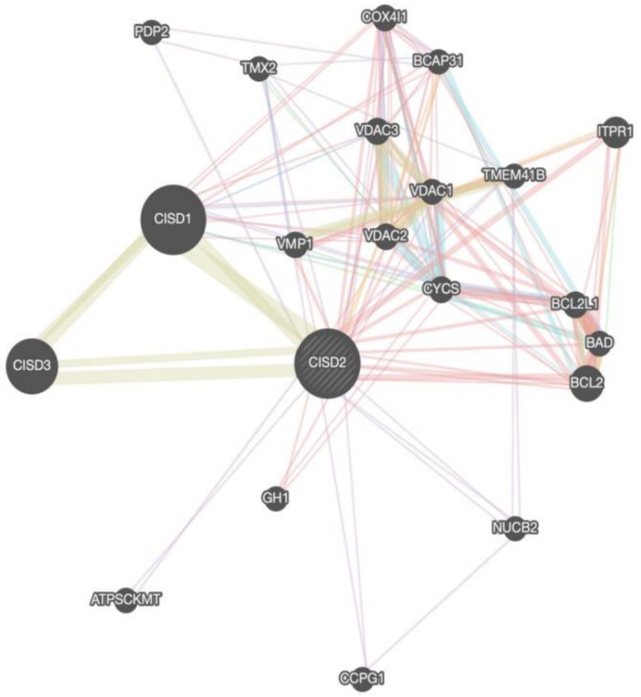

Figure S20. GeneMANIA gene network: CISD2 as the core.

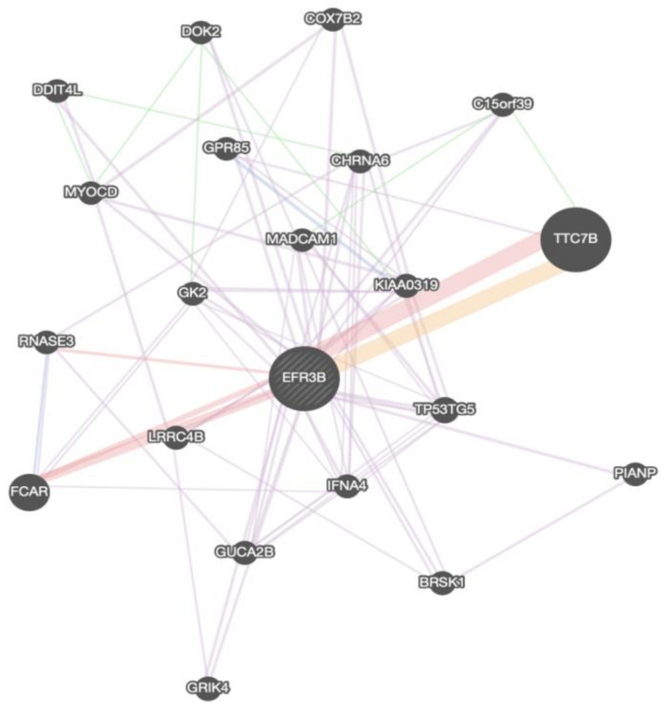

Figure S21. GeneMANIA gene network: EFR3B as the core.

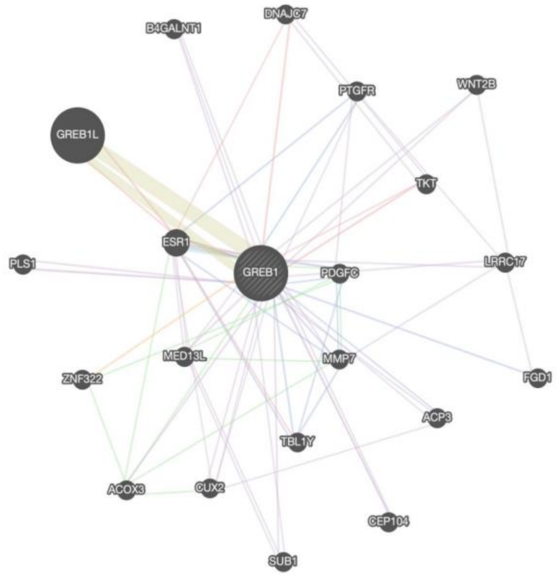

Figure S22. GeneMANIA gene network: GREB1 as the core.

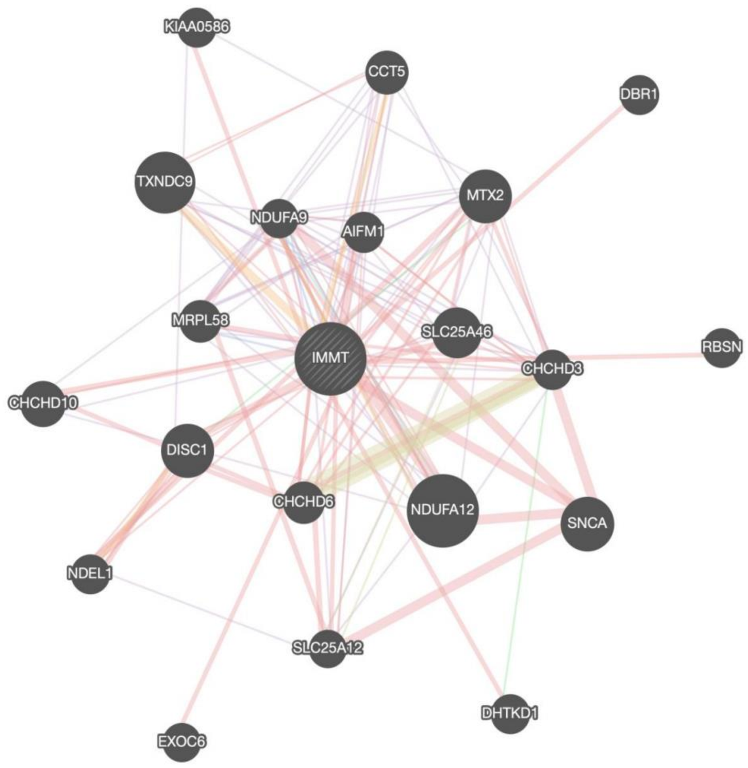

Figure S23. GeneMANIA gene network: IMMT as the core.

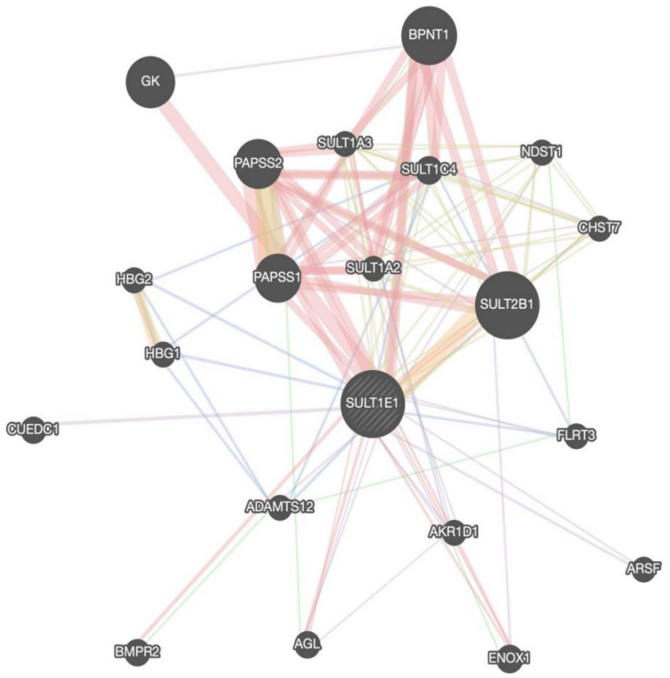

Figure S24. GeneMANIA gene network: SULT1E1 as the core.

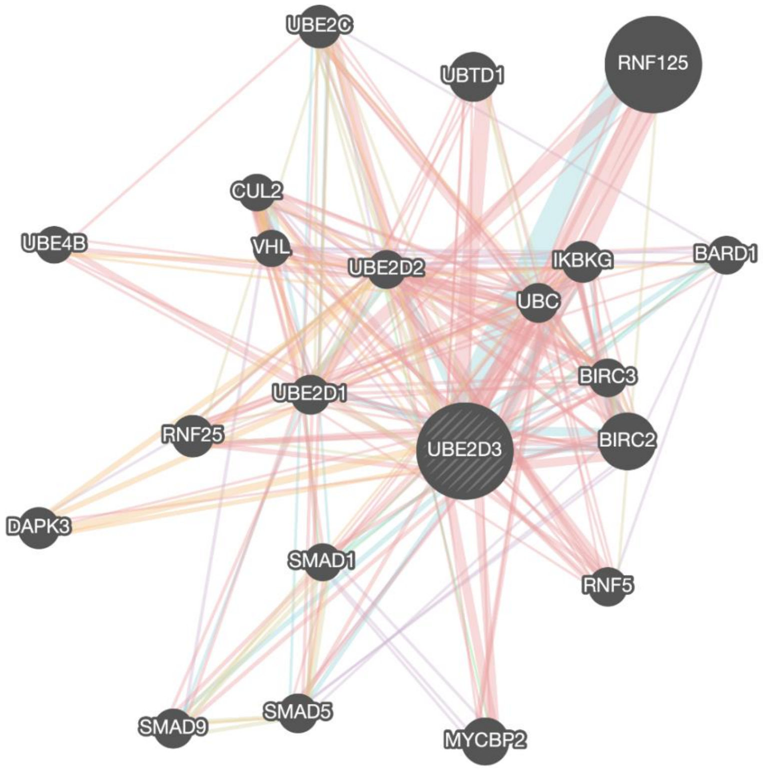

Figure S25. GeneMANIA gene network: UBE2D3 as the core.

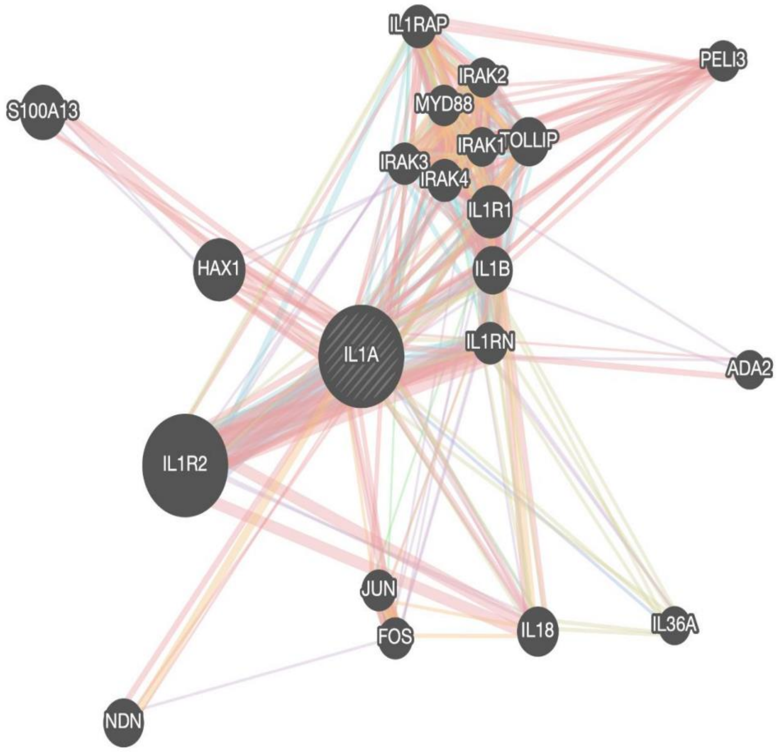

Figure S26. GeneMANIA gene network: IL1A as the core.

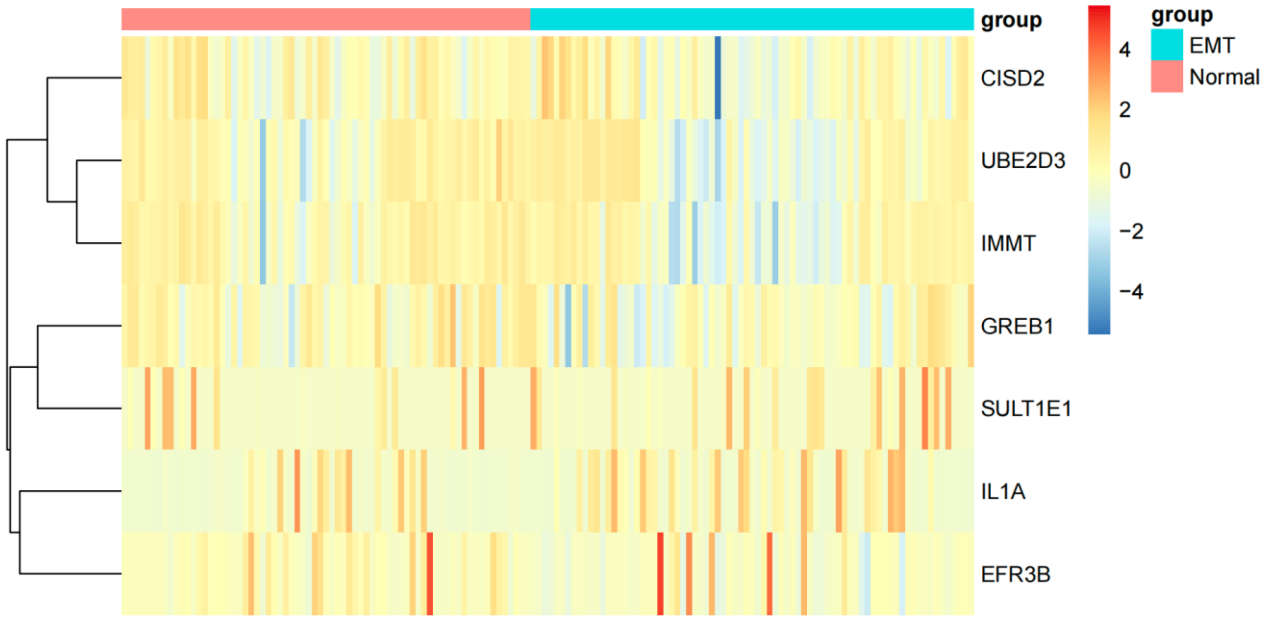

Figure S27. The heatmap of identified genes between EMT and control groups. Red and blue grids represent up- and down-regulated genes, respectively. CT, Control; EMT, Endometriosis.

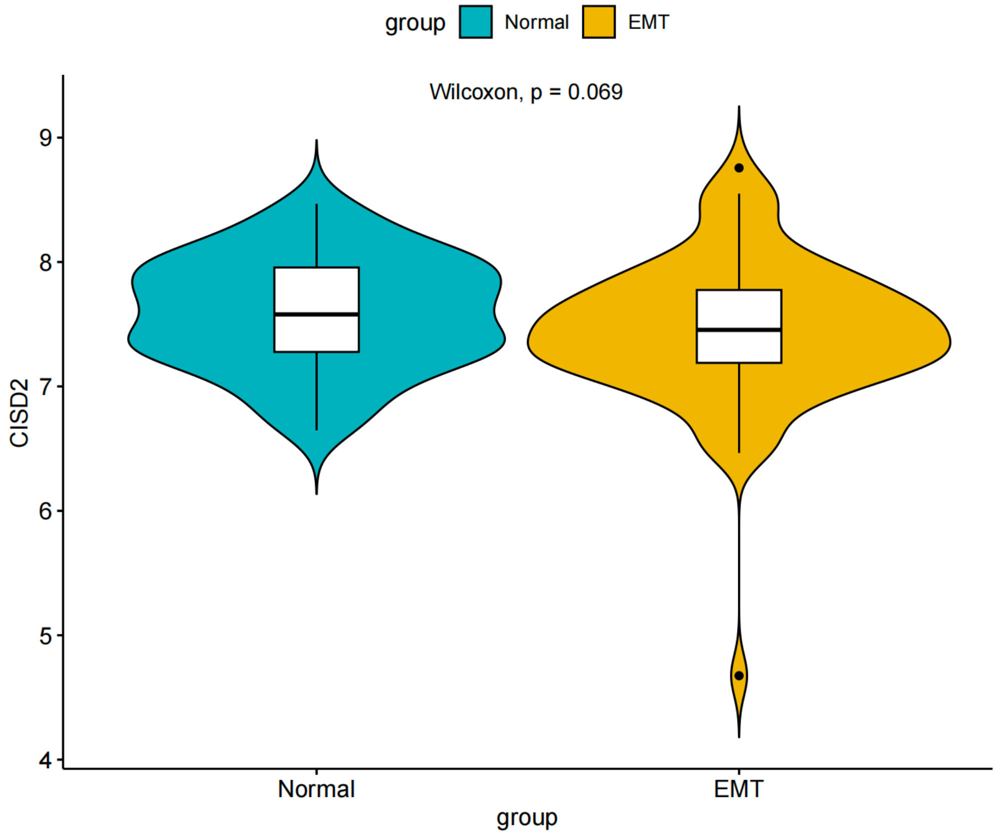

Figure S28. The expression differences of CISD2 between EMT group and CT group. CT, Control; EMT, Endometriosis.

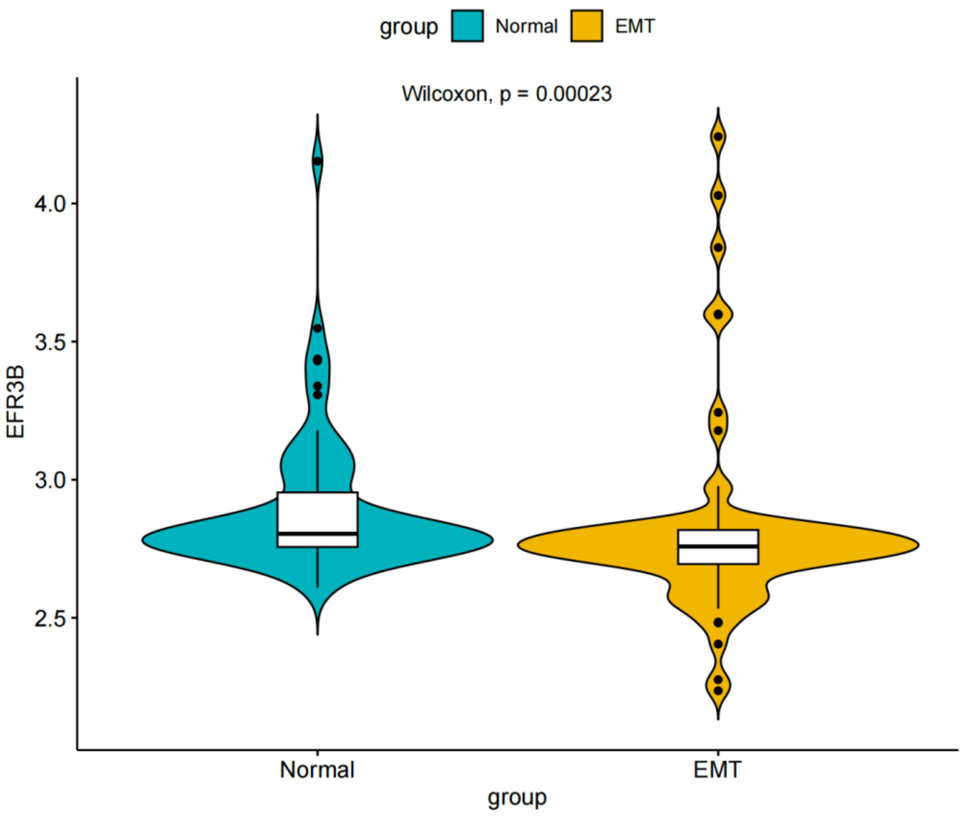

Figure S29. The expression differences of EFR3B between EMT group and CT group. CT, Control; EMT, Endometriosis.

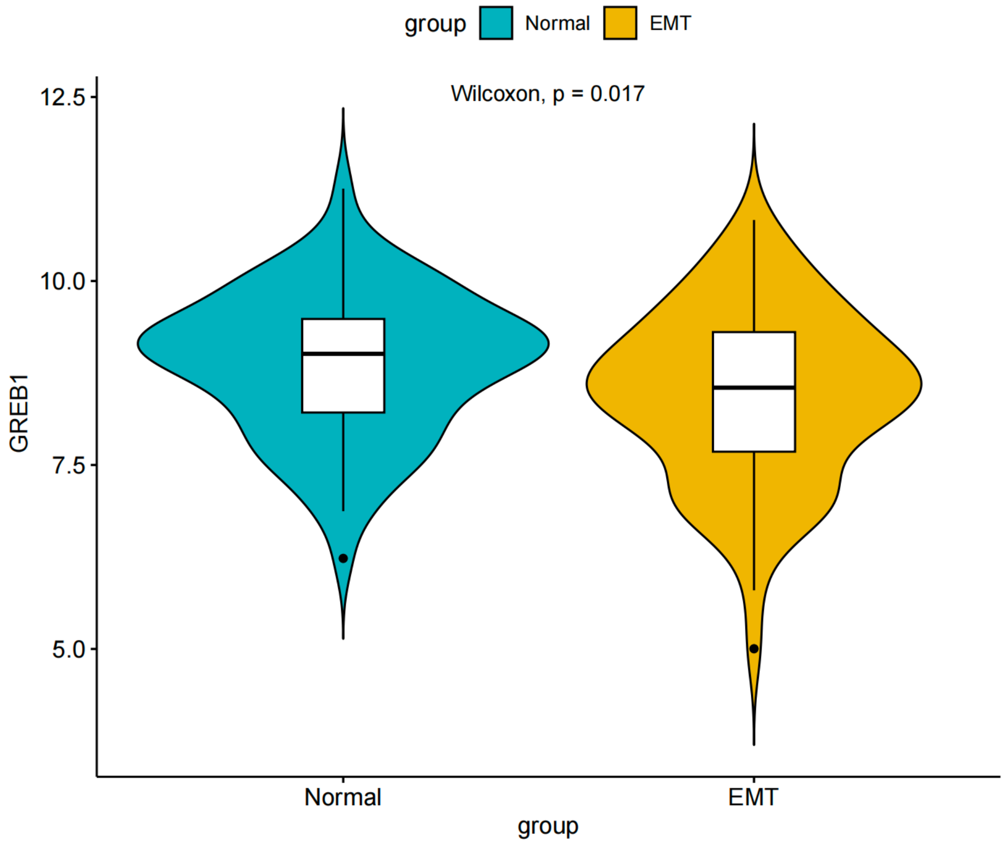

Figure S30. The expression differences of GREB1 between EMT group and CT group. CT, Control; EMT, Endometriosis.

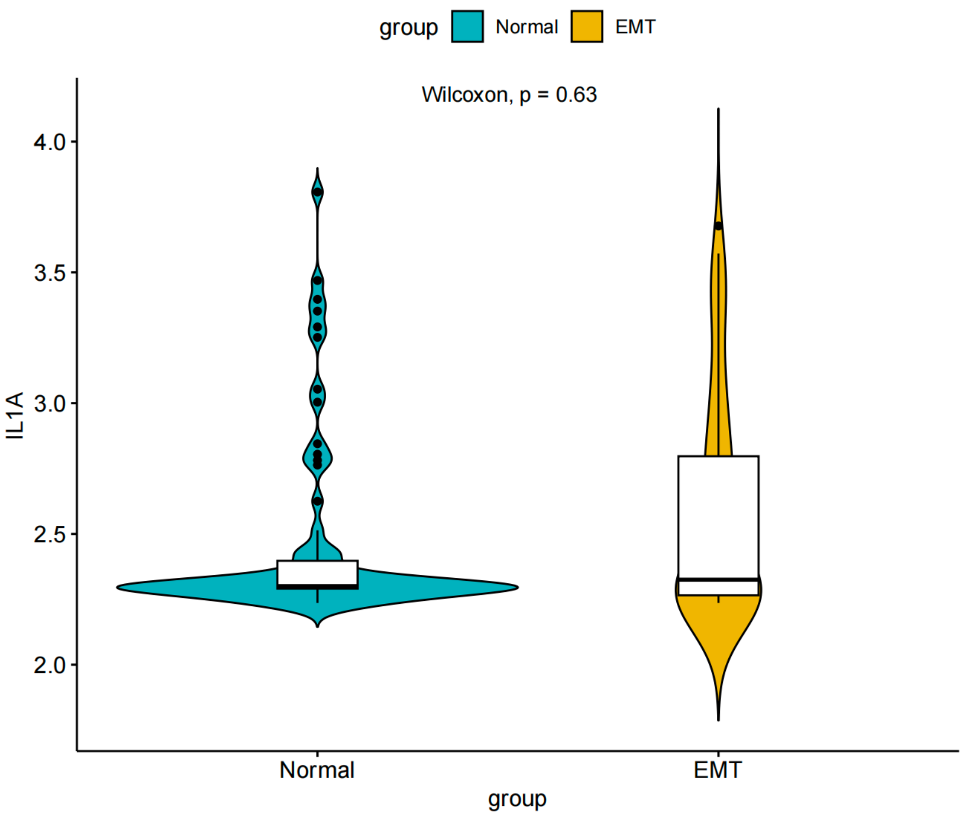

Figure S31. The expression differences of IL1A between EMT group and CT group. CT, Control; EMT, Endometriosis.

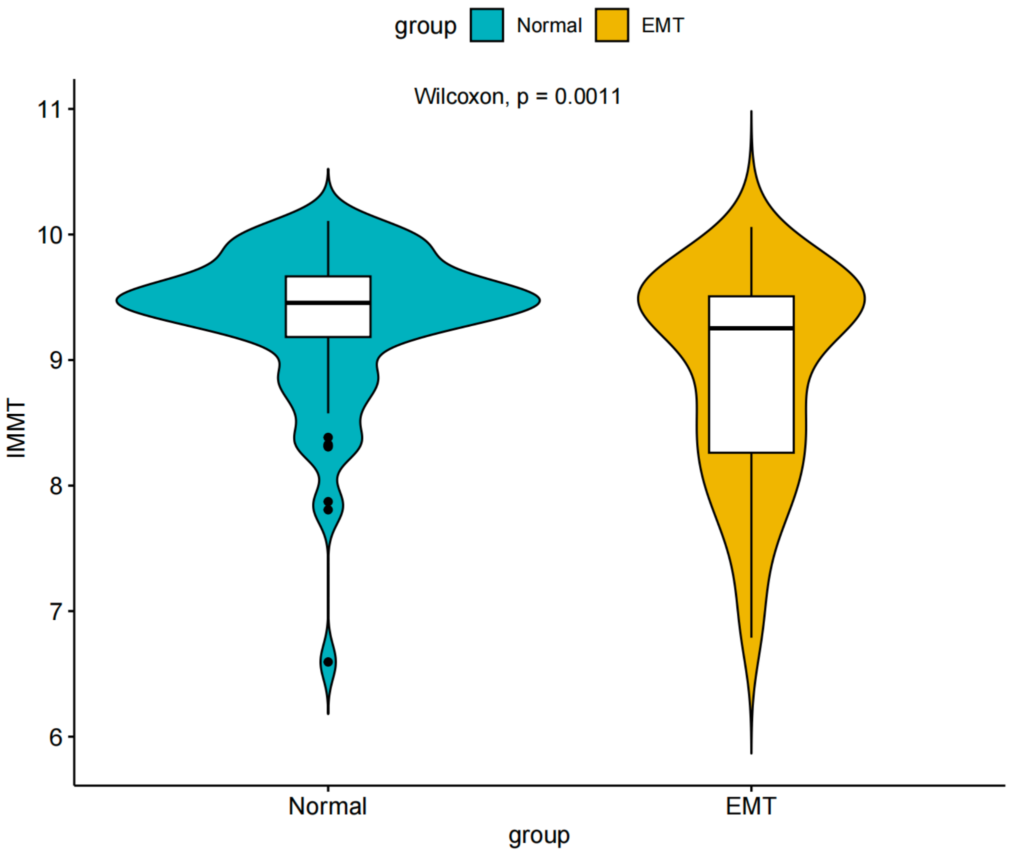

Figure S32. The expression differences of IMMT between EMT group and CT group. CT, Control; EMT, Endometriosis.

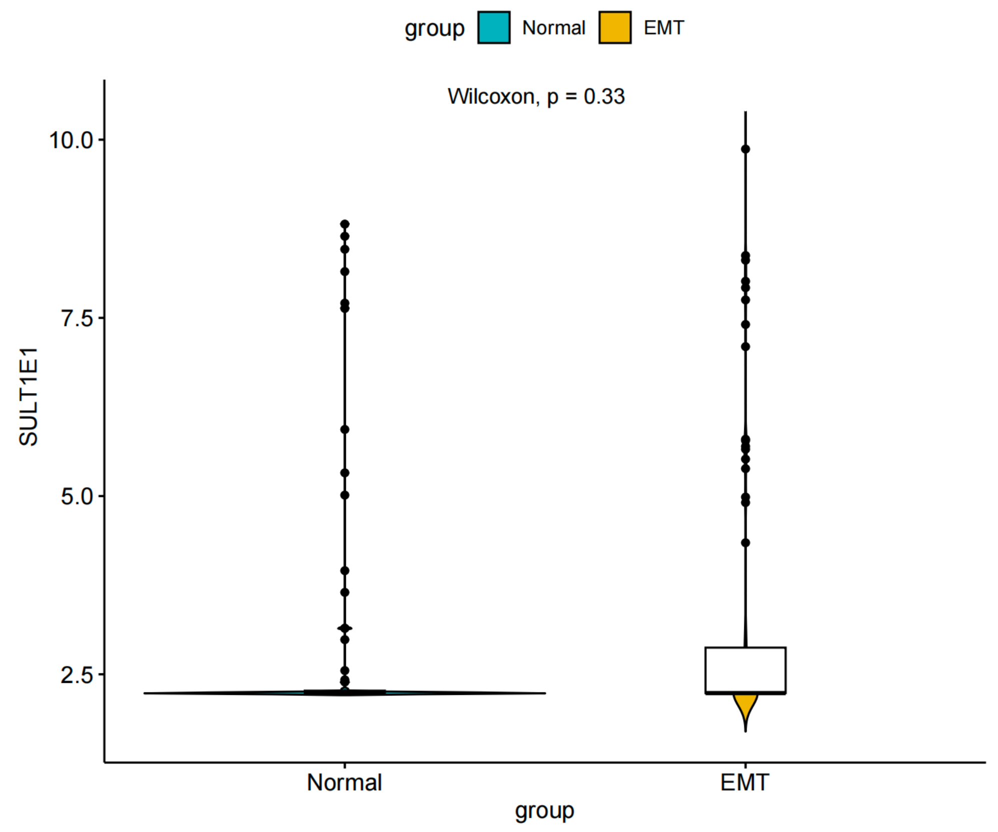

Figure S33. The expression differences of SULT1E1 between EMT group and CT group. CT, Control; EMT, Endometriosis.

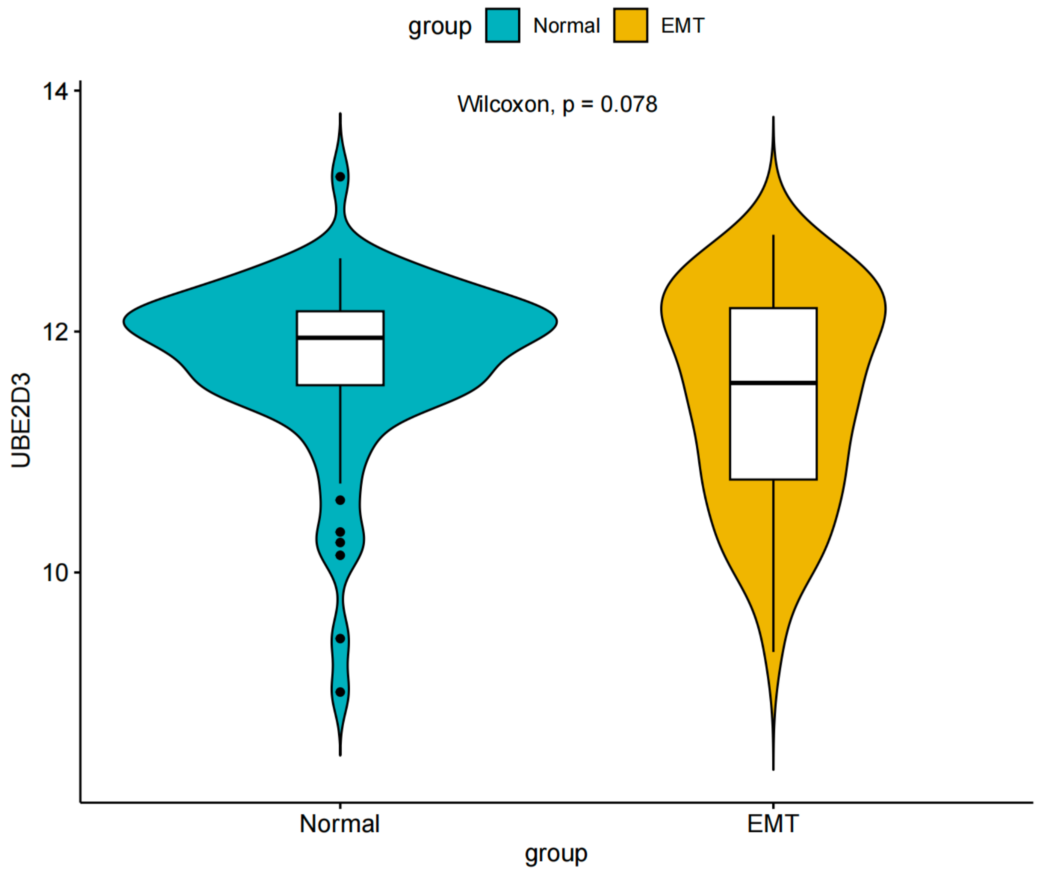

Figure S34. The expression differences of UBE2D3 between EMT group and CT group. CT, Control; EMT, Endometriosis.
